# Rearrangements involving 11q23.3/*KMT2A* in adult AML: mutational landscape and prognostic implications – a HARMONY study

**DOI:** 10.1038/s41375-024-02333-4

**Published:** 2024-07-04

**Authors:** Alberto Hernández-Sánchez, Teresa González, Marta Sobas, Eric Sträng, Gastone Castellani, María Abáigar, Peter J. M. Valk, Ángela Villaverde Ramiro, Axel Benner, Klaus H. Metzeler, Raúl Azibeiro, Jesse M. Tettero, Joaquín Martínez-López, Marta Pratcorona, Javier Martínez Elicegui, Ken I. Mills, Christian Thiede, Guillermo Sanz, Konstanze Döhner, Michael Heuser, Torsten Haferlach, Amin T. Turki, Dirk Reinhardt, Renate Schulze-Rath, Martje Barbus, Jesús María Hernández-Rivas, Brian Huntly, Gert Ossenkoppele, Hartmut Döhner, Lars Bullinger

**Affiliations:** 1 Hematology Department, University Hospital of Salamanca, Salamanca, Spain; 2 Institute of Biomedical Research of Salamanca (IBSAL), Salamanca, Spain; 3 Cancer Research Center of Salamanca (IBMCC, USAL-CSIC), Salamanca, Spain; 4 Wroclaw Medical University, Wroclaw, Poland; 5 Charité Universitätsmedizin Berlin, Berlin, Germany; 6 DIMES, University of Bologna, Bologna, Italy; 7 Department of Hematology, Erasmus MC Cancer Institute, University Medical Center Rotterdam, Rotterdam, The Netherlands; 8 Division of Biostatistics, German Cancer Research Center (DKFZ), Heidelberg, Germany; 9 University of Leipzig, Leipzig, Germany; 10 Department of Hematology, Amsterdam UMC Location VUMC, Amsterdam, The Netherlands; 11 Hospital Universitario 12 de Octubre, Madrid, Spain; 12 Department of Hematology, Hospital de la Santa Creu i Sant Pau, Barcelona, Spain; 13 Patrick G Johnston Centre for Cancer Research, Queen’s University, Belfast, UK; 14 University of Technics Dresden Medical Department, Dresden, Germany; 15 CIBERONC, Instituto de Salud Carlos III, Madrid, Spain; 16 Hospital Universitario y Politécnico La Fe, Valencia, Spain; 17 Department of Internal Medicine III, University Hospital of Ulm, Ulm, Germany; 18 Department of Hematology, Hemostasis, Oncology and Stem Cell Transplantation, Hannover Medical School, Hannover, Germany; 19 MLL Munich Leukemia Laboratory, Munich, Germany; 20 Marienhospital University Hospital, Ruhr-University Bochum, Bochum, Germany; 21 Universitätsklinikum Essen, Essen, Germany; 22 Department of Pediatrics III, University Hospital Essen, University Duisburg-Essen, Essen, Germany; 23 Bayer AG, Pharmaceuticals Division, Berlin, Germany; 24 AbbVie Deutschland GmbH & Co KG, Wiesbaden, Germany; 25 Department of Medicine, University of Salamanca, Salamanca, Spain; 26 Wellcome-MRC Cambridge Stem Cell Institute, University of Cambridge, Cambridge, UK; 27 Department of Hematology, Oncology, and Cancer Immunology, Charité – Universitätsmedizin Berlin, Berlin, Germany

**Keywords:** Acute myeloid leukaemia, Acute myeloid leukaemia

## Abstract

Balanced rearrangements involving the *KMT2A* gene (*KMT2A*r) are recurrent genetic abnormalities in acute myeloid leukemia (AML), but there is lack of consensus regarding the prognostic impact of different fusion partners. Moreover, prognostic implications of gene mutations co-occurring with *KMT2A*r are not established. From the HARMONY AML database 205 *KMT2A*r adult patients were selected, 185 of whom had mutational information by a panel-based next-generation sequencing analysis. Overall survival (OS) was similar across the different translocations, including t(9;11)(p21.3;q23.3)/*KMT2A*::*MLLT3* (p = 0.756). However, independent prognostic factors for OS in intensively treated patients were age >60 years (HR 2.1, p = 0.001), secondary AML (HR 2.2, p = 0.043), *DNMT3A*-mut (HR 2.1, p = 0.047) and *KRAS*-mut (HR 2.0, p = 0.005). In the subset of patients with de novo AML < 60 years, *KRAS* and *TP53* were the prognostically most relevant mutated genes, as patients with a mutation of any of those two genes had a lower complete remission rate (50% vs 86%, p < 0.001) and inferior OS (median 7 vs 30 months, p < 0.001). Allogeneic hematopoietic stem cell transplantation in first complete remission was able to improve OS (p = 0.003). Our study highlights the importance of the mutational patterns in adult *KMT2A*r AML and provides new insights into more accurate prognostic stratification of these patients.

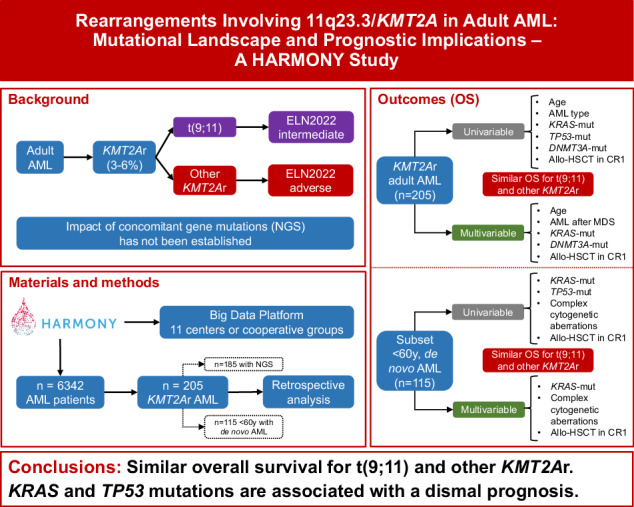

## Introduction

Acute myeloid leukemia (AML) is a heterogeneous disease in terms of clinical features and outcomes, in which the presence of cytogenetic aberrations and gene mutations provides crucial prognostic information that guides clinical decisions [1–5]. Balanced rearrangements involving the *lysine methyltransferase 2a* gene (*KMT2A*, previously known as *MLL*), located on chromosome band 11q23.3, have been described in 3–6% of adult patients with de novo AML [6–11].

Balanced chromosome rearrangements involving 11q23.3 and *KMT2A* (*KMT2A*r) are very heterogeneous, as more that 100 fusion partners have already been described in leukemia patients [12]. Among them, the most frequent is the t(9;11)(p21.3;q23.3) resulting in a *KMT2A::MLLT3* fusion, which will be referred to as t(9;11) hereafter. Other common translocations in adult AML are the t(6;11)(q27;q23.3)/*KMT2A::AFDN* [hereafter t(6;11)], t(11;19)(q23.3;p13.3)/*KMT2A::MLLT1*, t(11;19)(q23.3;p13.1) resulting in a *KMT2A::ELL* fusion, t/ins(10;11)(p12.3;q23.3)/*KMT2A::MLLT10* [hereafter t(10;11)], and t(11;17)(q23.3;q25) resulting in a *KMT2A::SEPTIN9* fusion [hereafter t(11;17)] [7, 13, 14].

There is a controversy in the literature regarding the prognostic impact of different *KMT2A*r AML. Several studies have reported better outcomes in patients with t(9;11) when compared to the rest of *KMT2A*r [7, 14–16], although some of them were restricted to specific populations (e.g., patients aged <60 years with de novo AML). On the other hand, these findings could not be confirmed by other studies [6, 17–19]. Moreover, heterogeneous results have also been reported regarding outcome in the post-allogeneic transplantation setting [20–22].

Additional cytogenetic aberrations have been identified in about 40% of *KMT2A*r adult AML patients, but they were not associated with different patient outcomes [11, 16, 17]. While distinct gene mutations have proven to be excellent prognostic markers in AML [1–4, 23], *KMT2A*r AML shows a lower incidence of co-occurring gene mutations, although a predominance of *RAS* mutations has been reported. The prognostic impact of these additional gene mutations in *KMT2A*r adult AML has not been established yet [9, 14, 17, 19].

In order to address these open questions, we analyzed a large cohort of patients with *KMT2A*r AML included in the Healthcare Alliance for Resourceful Medicine Offensive against Neoplasms in Hematology (HARMONY) AML multicenter database.

## Methods

### Patients

At the time of the analysis (December 2022) the HARMONY Alliance AML database contained 6342 AML patient data sets contributed by 11 European centers or cooperative groups (Supplementary Table S1). Conventional karyotype information was available for 6005 patients based on which a total of 205 *KMT2A*r adult patients could be identified (incidence of *KMT2A*r: 3.4%), who were diagnosed with AML between December 1996 and January 2020 (Supplementary Fig. S1). Some of the patients included in this analysis were also part of previously published AML cohorts [3, 9, 24–26].

For data upload into the HARMONY Big Data Platform, all patient data went through a robust double brokerage pseudonymization procedure in compliance with the General Data Protection Regulation. Next, data were harmonized and transformed using the Observational Medical Outcomes Partnership (OMOP) Common Data Model [27].

This study was performed in accordance with the Declaration of Helsinki and was approved by the HARMONY steering committee. The HARMONY research project was reviewed and approved by the Medicinal Research Ethics Committee of the University of Salamanca. For its studies, HARMONY provides an ethical and data-protection framework for the secondary use of data including a *de facto* anonymization. Written informed consent had been previously collected from all patients in the respective HARMONY partner institutions.

### Cytogenetic and genetic analyses

Cytogenetic analyses were performed using standard G-banding with trypsin-Giemsa or trypsin-Wright staining in approved cytogenetic laboratories. The analysis and nomenclature of the chromosomes were based on International System for Human Cytogenetic Nomenclature (ISCN) [28]. Karyotypes were considered with clonal abnormalities if at least two metaphases showing the same abnormality were detected. The results of cytogenetic analysis of bone marrow were centrally reviewed by experienced cytogeneticists (JMHR and TGM) using standard ISCN-2020 criteria. Although t(11;19)(q23.3;p13.3) and t(11;19)(q23.3;p13.1) are known to be two distinct rearrangements [13], enough information to discriminate was only provided for around half of those patients, so we grouped them together as t(11;19)(q23;p13) [hereafter t(11;19)].

*KMT2A*r cases were also evaluated by fluorescence in situ hybridization (FISH) with a commercial break apart probe in order to confirm *KMT2A*r. FISH results were also centrally reviewed and reported using standard ISCN-2020 nomenclature. For selected cases, the *AF9*::*KMT2A* fusion transcript was also validated by reverse transcription polymerase chain reaction (RT-PCR) using the generic PCR cycler program developed by the BIOMED-1 initiative for the standardized RT-PCR analysis of fusion gene transcripts from chromosome aberrations in acute leukemia [29].

Next-generation sequencing (NGS) data using local custom diagnostic panels were available for 185 patients (90% of our *KMT2A*r cohort). The different NGS panels overlapped with regard to 40 genes implicated in myeloid malignancies (Supplementary Table S2). Panel sequencing was performed and analyzed according to previous studies [3, 9, 24]. Gene variants were centrally reviewed, only pathogenic and likely pathogenic variants were considered, and data including variant allele frequency were harmonized.

### Statistical analysis

Clinical endpoints were defined as recommended by international guidelines [4]. Composite complete remission (CRc) was defined as either complete remission (CR) or CR with incomplete hematologic recovery (CRi). Categorical variables were compared using Pearson’s Chi-squared test and Fisher’s exact test, while continuous variables were evaluated using Kruskal–Wallis test for multiple comparisons and Wilcoxon test for pairwise comparison. Overall survival (OS) and relapse-free survival (RFS) were estimated using the Kaplan–Meier method and differences between survival distributions were evaluated using the log-rank test. To estimate survival probabilities considering the effect of allogeneic hematopoietic stem cell transplantation (allo-HSCT) in first complete remission (CR1), Simon–Makuch method with clock-back correction was applied as previously reported [30, 31]. Cox proportional hazards model was used for multivariate survival analysis, including variables that were significant in the univariate analysis (age, AML type, *KRAS*-mut, *DNMT3A*-mut, *TP53*-mut and presence of complex cytogenetic aberrations), as well as *KMT2A* fusion partner. To examine the effect of allo-HSCT in CR1, multivariate Cox model with allo-HSCT as a time-depending intervening event was used. All reported p values are two-sided at the conventional 5% significance level. Data were analyzed as of December 2022 using R software (v3.6.3).

## Results

### Patient characteristics

The study population of 205 *KMT2A*r adult AML patients included 54% females and median age at diagnosis was 48 years (range 18–86), while 75% of the patients were younger than 60 years. Most of the patients (73%) had de novo AML and 43% underwent allogeneic hematopoietic stem cell transplantation (allo-HSCT). Median follow-up was 4.9 years for those patients still alive. Patient baseline characteristics are summarized in Table 1 and Supplementary Table S3.

**Table 1:** Baseline characteristics of *KMT2A*r adult AML patients.

	n = 205
Female sex	111 (54.1%)
Median age in years (range)	48.1 (18.6–86.2)
Age ≥ 60 years	51 (24.9%)
AML type	
De novo AML	148 (72.2%)
Secondary AML	57 (27.8%)
AML after MDS	14 (6.8%)
Therapy-related AML	43 (21%)
Hemoglobin (g/dL)	9.5 [Q1 = 8.3, Q3 = 11.3]
WBC (×10^9^/L)	23.1 [Q1 = 4.7, Q3 = 58]
(>100 × 10^9^/L)	21 (10.2%)
Platelets (×10^9^/L)	51.5 [Q1 = 29.8, Q3 = 107.5]
Bone marrow % of blasts	83 [Q1 = 70.2, Q3 = 90]
Chromosomal aberrations	
t(9;11)	101 (49%)
t(11;19)	33 (16%)
t(6;11)	24 (12%)
t(10;11)	10 (5%)
t(11;17)	10 (5%)
Other *KMT2A*r	27 (13%)
Additional chromosomal aberrations	85 (41.6%)
Complex aberrations (≥2 apart from *KMT2A*r)	38 (19%)
Trisomy 8	37 (18%)
Derivative 11	12 (6%)
ELN 2022	
Favorable	–
Intermediate	101 (49.3%)
Adverse	104 (50.7%)
Treatment	
Intensive	195 (95%)
Non-intensive	10 (5%)
Composite complete response	120 (63.8%)
Early death	
30-day mortality	28 (13.6%)
60-day mortality	36 (17.6%)
Allogeneic HSCT	88 (42.9%)
In CR1	48 (23.4%)
In CR2 or later	15 (7.3%)
Unknown response at allo-HSCT	25 (12.2%)
Median survival in years (95% CI)	1.4 (1.1–1.7)

*AML* acute myeloid leukemia, *MDS* myelodysplastic syndrome, *WBC* white blood cell, *ELN* European LeukemiaNet, *HSCT* hematopoietic stem cell transplantation, *CR1* first complete remission, *CR2* second complete remission, *CI* confidence interval.

### Frequency of specific 11q23.3/*KMT2A* rearrangements

Translocation t(9;11) was the most frequent *KMT2A*r [n = 101, 49%], followed by t(11;19) [n = 33, 16%] and t(6;11) [n = 24, 12%]. Both t(10;11) and t(11;17) accounted for 5% of patients each (n = 10), while the less common translocations grouped together (hereafter “other *KMT2A*r”) represented 13% of patients. A comparison of basic characteristics among different translocation partners showed differences in age distribution, with the youngest patients being in t(6;11) [median 41 years] and the oldest in the “other *KMT2A*r” subgroup (median 55.9 years) (p = 0.004) (Supplementary Table S4). In addition, the proportion of therapy-related AML was highest in the t(9;11) [30.7%] subgroup, while none was found in the t(6;11) cases (p = 0.013). Allo-HSCT rates did not differ significantly among distinct *KMT2A*r categories (p = 0.523).

### Additional cytogenetic abnormalities

Additional cytogenetic abnormalities were present in 40% of patients. Complex aberrations (≥2 abnormalities in addition to the *KMT2A*r) were the most frequent (19%), followed by trisomy 8 (18%), derivative 11 (6%) and trisomy 21 (5%). Isolated trisomy 8 as the only additional cytogenetic abnormality (apart from *KMT2A*r) was found in 19 patients (9%) and was almost exclusively found in the t(9;11) subgroup (16 patients, p < 0.001). On the other hand, complex aberrations were more frequently associated with “other *KMT2A*r” (p = 0.001).

### Mutational landscape

NGS analysis was performed in 185 (90%) cases using a panel of up to 90 genes (Supplementary Table S2). A total of 263 mutations were found in 44 of the 90 analyzed genes. The median number of gene mutations per patient was 1 (range 0–6).

**Fig. 1:**
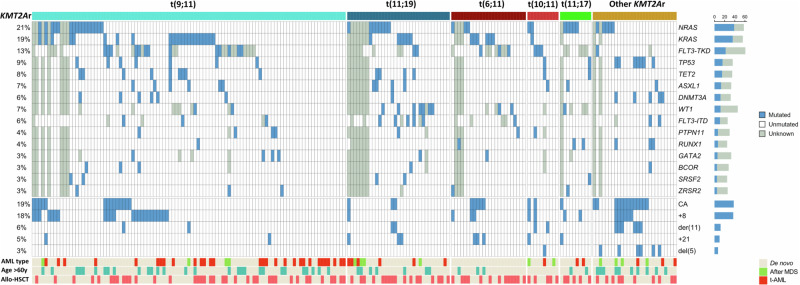
Mutational landscape and additional cytogenetic aberrations in *KMT2A*r adult AML by fusion partner. Only mutations present in at least five patients and most frequent cytogenetic aberrations are shown. Allo-HSCT allogeneic hematopoietic stem cell transplantation; CA complex cytogenetic aberrations (≥2 apart from *KMT2A*r); MDS myelodysplastic syndrome; t-AML therapy-related acute myeloid leukemia.

The most frequent gene mutations co-occurring with a *KMT2A*r were *NRAS* (21%), *KRAS* (19.5%) and *FLT3*-TKD (13.3%), followed by *TP53* (8.6%), *TET2* (8.1%), *ASXL1* (7%), *WT1* (7%), *DNTM3A* (6.5%) and *FLT3*-ITD (5.8%). When grouped together, mutations in genes that comprise the RAS signaling pathway (*NRAS*, *KRAS, PTPN11* and *BRAF*) were present in 42.1% of *KMT2A*r AML. Of note, *NRAS* and *KRAS* mutations were not mutually exclusive in our cohort, as seven patients harbored mutations in both genes. The distribution of mutations within the different *KMT2Ar* subgroups is shown in Fig. 1. *WT1* mutation was predominant in t(11;19) subgroup (p < 0.001), while *TP53* mutation was recurrent in the “other *KMT2A*r” subgroup (p = 0.002).

### Patient outcomes

**Fig. 2:**
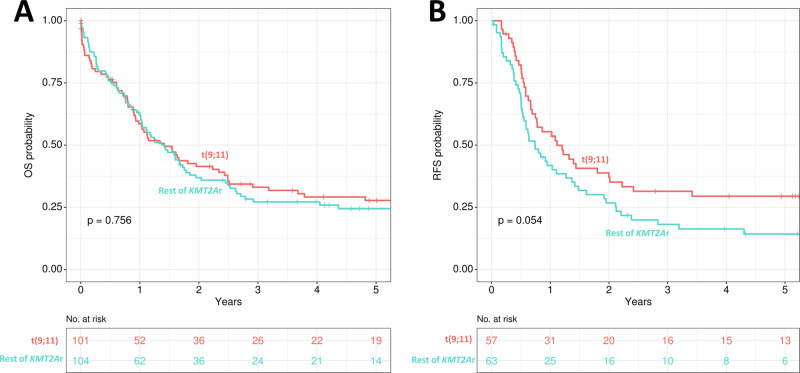
Comparison of survival outcomes between t(9;11) and the rest of the *KMT2A*r cohort. **A** Overall survival. **B** Relapse-free survival.

Median OS was 1.4 years for the whole cohort. Notably, OS was similar across the different *KMT2Ar* subgroups and we were not able to find any differences in OS between t(9;11) and the remainder of the *KMT2Ar* patients (p = 0.756, Fig. 2A). However, in t(9;11) patients there was a trend for better RFS (p = 0.054, Fig. 2B).

Patients harboring concomitant cytogenetic aberrations had a higher rate of early deaths (first 60 days 23.2% vs 7.3%, p = 0.002), although those patients were older (median age 52 vs 46 years, p = 0.008) and median overall survival was similar to the rest of the cohort (12 vs 15 months, p = 0.529), as shown in Supplementary Fig. S2A. Complex aberrations (≥2 additional cytogenetic abnormalities) were associated with shorter OS, with median OS of 9 vs 18 months for the rest of the cohort (p = 0.046, Supplementary Fig. S2B). Otherwise, we did not find significant differences in OS between patients with trisomy 8 when compared to the remainder of patients (p = 0.920, Supplementary Fig. S2C). Patients with isolated trisomy 8 as the only additional cytogenetic aberration also presented with similar OS compared to the rest of the cohort (p = 0.402, Supplementary Fig. S2D).

**Fig. 3:**
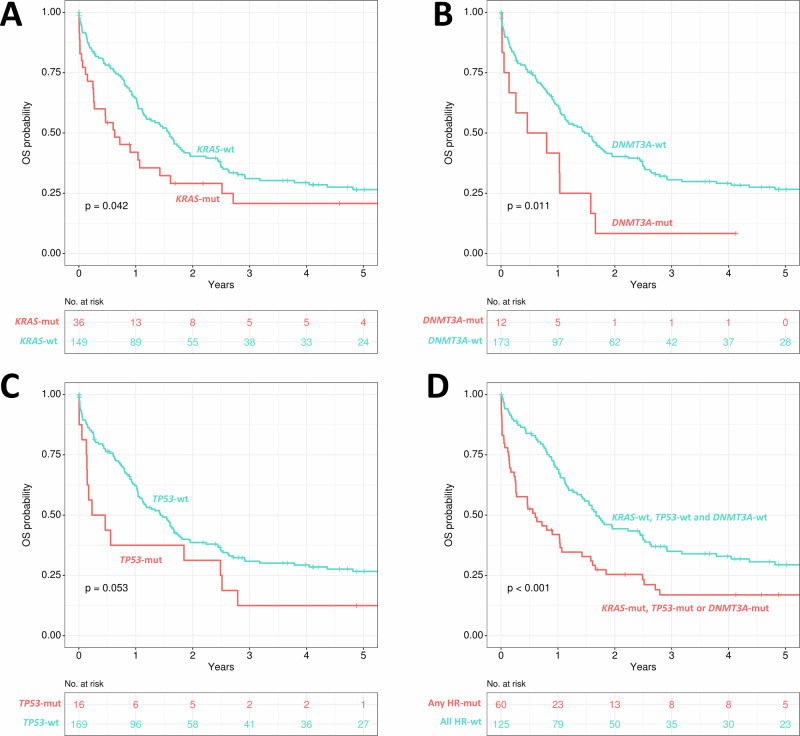
Impact of different gene mutations on overall survival in *KMT2A*r AML. **A** *KRAS*, **B** *DNMT3A*, **C** *TP53*, **D** comparison of patients with either *KRAS*-mut, *DNMT3A*-mut or *TP53*-mut and the rest of the *KMT2A*r AML cohort; OS overall survival; mut mutated; wt wildtype.

Given that NGS information on mutational landscape was available for the vast majority of the patients of the cohort (90%, n = 185), the impact of gene mutations on OS was further analyzed. *KRAS* mutations were present in 36 patients and were associated with shorter OS, with a median OS of 8 vs 19 months for *KRAS*-mut vs *KRAS*-wt patients (p = 0.042, Fig. 3A). *NRAS*-mut was the most frequent mutation (39 patients), but it was not associated with shorter OS (p = 0.883, Supplementary Fig. S3A) and only patients presenting both mutations (*NRAS* and *KRAS*, 7 patients) had reduced survival when compared to the rest of the cohort (p = 0.041, Supplementary Fig. S3B). Patients with *DNMT3A*-mut also presented with shorter OS with a median OS of 8 vs 18 months for *DNMT3A*-mut vs *DNMT3A*-wt patients (p = 0.011, Fig. 3B), and there was a trend for poor outcome in *TP53*-mut patients (p = 0.053, Fig. 3C).

**Fig. 4:**
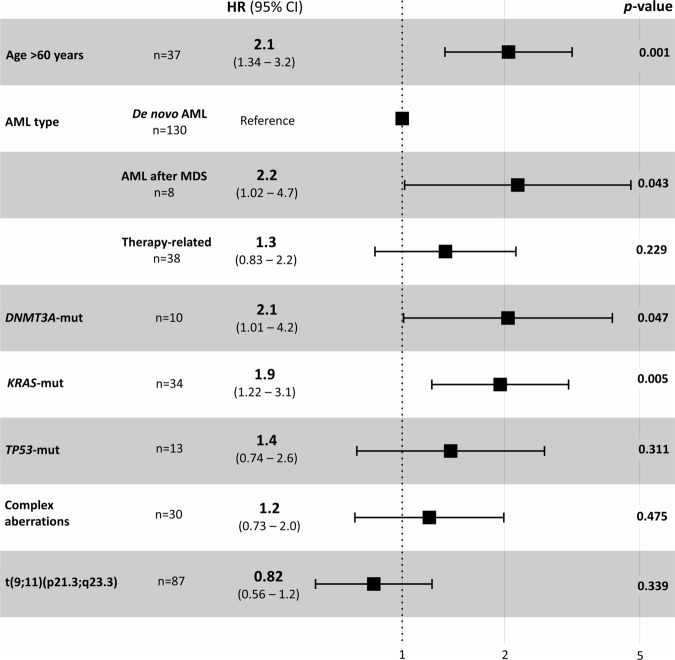
Multivariate Cox regression model for prediction of overall survival in intensively treated patients. HR hazard ratio; CI confidence interval; AML acute myeloid leukemia; MDS myelodysplastic syndrome; mut mutated.

Patients harboring a mutation of any of the above-mentioned high-risk genes (either *KRAS*, n = 36; *DNMT3A*, n = 12 or *TP53*, n = 16) represented 32% of the cohort and had a median OS of 7 months, while the rest of the patients had a median OS of 20 months (p < 0.001, Fig. 3D). A multivariate Cox regression model for intensively treated patients identified the following independent pretreatment variables regarding OS: age > 60 years (HR 2.1, p = 0.001), secondary AML after MDS (HR 2.2, p = 0.043), *DNMT3A*-mut (HR 2.1, p = 0.047), *KRAS*-mut (HR 2.0, p = 0.005), as shown in Fig. 4. Of note, therapy-related AML, complex cytogenetic aberrations, t(9;11) or *TP53*-mut were not significant in this model.

### Effect of allo-HSCT in *KMT2A*r AML

Allo-HSCT was performed in 88 patients (42.9%), at CR1 in 48 (23.4%) and CR2 or later in 15 (7.3%), while the response at allo-HSCT was not known in 25 (12.2%). The estimated survival probabilities using Simon–Makuch method with clock-back correction revealed a significant OS benefit of allo-HSCT in CR1 (p = 0.003, Supplementary Fig. S4A). Moreover, patients with *KRAS*-mut, *TP53*-mut or *DNMT3A*-mut AML also benefited from allo-HSCT in CR1 (p = 0.026, Supplementary Fig. S4B). Remarkably, from all patients who underwent allo-HSCT in CR1, OS for *KRAS*-mut, *TP53*-mut or *DNMT3A*-mut patients was similar to those patients lacking these mutations (p = 0.530, Supplementary Fig. S4C), as well as RFS (p = 0.884, Supplementary Fig. S4D). Allo-HSCT in CR1 was an independent prognostic variable for increased OS in a multivariate Cox regression model that included the same variables stated previously (p < 0.001, Supplementary Fig. S5).

### Outcome in intensively treated younger (<60 years) *KMT2A*r de novo AML patients

As the general cohort of 205 patients was heterogeneous in terms of age, origin of AML and treatment, an analysis in a subgroup of 115 patients aged <60 years at diagnosis, who received intensive chemotherapy regimens and who presented with de novo *KMT2A*r AML (excluding both therapy-related and AML after MDS) was carried out. Median age of this subgroup was 42 years (range 19–59) and it included 51% females. Median follow-up for those patients alive was 5.1 year, CRc rate was 75.7%, allo-HSCT was performed in 53.9% and median OS was 1.7 years.

Notably, OS was still similar across the different translocation partners, without significant differences in OS and similar RFS between t(9;11) and the remainder of the patients (p = 0.916, Supplementary Fig. S6A, and p = 0.193, Supplementary Fig. S6B, respectively). Interestingly, there was a trend for longer OS for patients who harbored isolated trisomy 8 (n = 10) as the only additional cytogenetic abnormality (p = 0.072, Supplementary Fig. S7A). In fact, 80% of the patients achieving a CRc had long-lasting responses with a 2-year RFS of 75% vs 31% for the rest of cases (p = 0.016, Supplementary Fig. S7B).

**Fig. 5:**
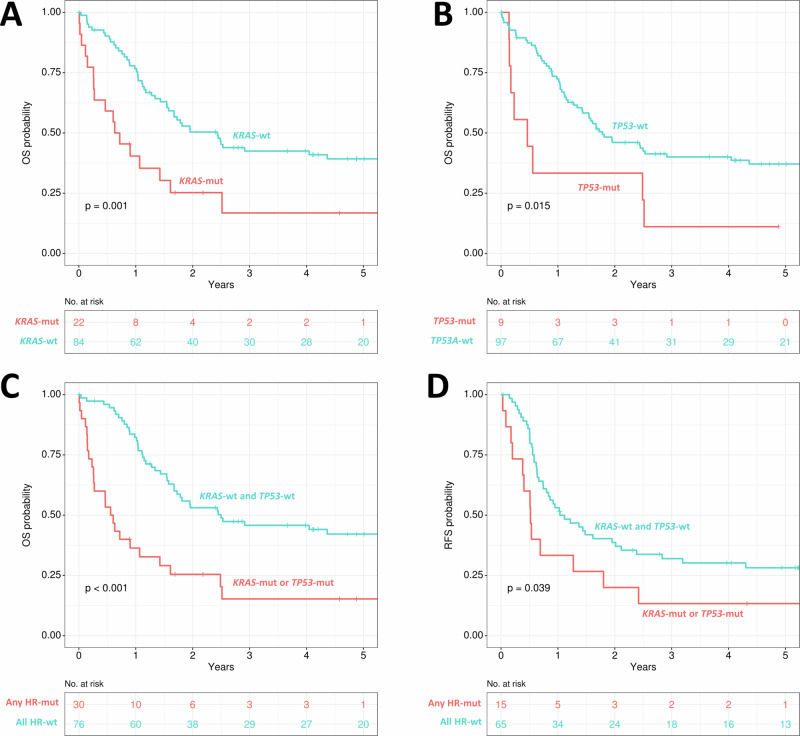
Impact of different gene mutations on overall survival in patients with de novo *KMT2A*r AML and aged ≤60 years. **A** *KRAS*, **B** *TP53*, **C** comparison of overall survival of patients with any high-risk genes mutated (either *KRAS*-mut, or *TP53*-mut) and the rest of patients aged ≤60 years with de novo AML, **D** comparison of relapse-free survival of patients with any high-risk mutated genes (either *KRAS*-mut or *TP53*-mut) and the rest of patients aged ≤60 years with de novo AML. OS overall survival; RFS relapse-free survival; mut mutated; wt wildtype; HR high-risk genes (*KRAS*, *TP53*).

Regarding gene mutations, patients with *KRAS*-mut (n = 22) had a significantly shorter OS (p = 0.001, Fig. 5A) when compared to *KRAS*-wt (n = 84). Patients showing *TP53*-mut (n = 9) were also associated with shorter OS (p = 0.015, Fig. 5B), while *DNMT3A-*mut was not significant (p = 0.242), although there was also a low number of patients who presented with *DNMT3A-*mut (n = 5) in this subset of younger AML patients (<60 years of age).

Therefore, *KRAS* and *TP53* were the most relevant genes linked to OS in this subgroup of patients. Grouped together (either *KRAS*-mut or *TP53*-mut) these mutations defined 28% (n = 30) of the cohort to have an adverse prognosis. These patients showed a significantly lower rate of CRc (50% vs 85.5%, p < 0.001). Median OS was 7 months and 5-year OS only 15%, while for the rest of patients median OS was 30 months and 5-year OS 42% (p < 0.001, Fig. 5C). Moreover, median RFS was 6 months for patients with *KRAS*-mut or *TP53*-mut, while median RFS was 13 months in all other younger *KMT2A*r cases (p = 0.039, Fig. 5D).

A multivariate Cox regression in this subset of patients identified the following independent variables regarding OS: *KRAS*-mut (HR 2.6, p = 0.002), complex cytogenetic aberrations (HR 2.3, p = 0.019), and allo-HSCT in CR1 (HR 0.7, p = 0.003), while there was a trend for *TP53*-mut (HR 2.2, p = 0.077), as shown in Supplementary Fig. S8.

### Historical comparison of outcomes in *KMT2A*r AML

As the cohort was accrued over several decades, the impact of the year of AML diagnosis on OS was also analyzed. As we have reported improved patient outcomes for patients treated in the decade between 2007 and 2016 when compared to the decade between 1997 and 2006 in the HARMONY AML database [32] the same cut-off was used for this analysis. However, we could not find differences in OS when stratifying by the year of diagnosis (p = 0.458, Supplementary Fig. S9A). In the subset of de novo AML patients aged <60 years, early-death rates were similar with 10% for the 1996–2006 period and 6.2% for the 2007–2020 period (p = 0.508) and again without statistical differences in OS (median OS 19 vs 24 months respectively, p = 0.396, Supplementary Fig. S9B).

## Discussion

*KMT2A*r are recognized as recurrent genetic abnormalities in AML, although they are present only in a minority of adult AML patients [4, 5, 23]. In the HARMONY AML database, we found an incidence of 3.4% (205 out of 6005) for *KMT2A*r AML, which is in line with previous studies [6, 7, 11, 15, 19]. This highlights the importance of large databases in order to obtain a representative cohort of *KMT2A*r adult AML. However, based on conventional diagnostics the incidence of *KMT2A*r AML might be slightly underestimated, as the development of molecular techniques has allowed the identification of cryptic *KMT2A* fusions, and thus the true prevalence might be higher [12]. While the incidence of *KMT2A*r AML was recently shown to be significantly higher in patients <60 years, the median age of 48 years in our cohort was well within the range of previous reports [6, 7, 14, 15, 17, 19].

The prognostic impact of different translocation partners in *KMT2A*r AML has been studied for several decades, with quite heterogeneous results [6, 7, 14–19]. Recently, Bill et al. [14] observed that the prognostic significance of t(9;11) was dependent on patient age, as it was associated with better outcomes only for patients aged <60 years with de novo AML. However, we could not corroborate that finding when we analyzed the same subset of patients. Interestingly, Bill et al. reported an uneven distribution of *KRAS* mutations among the different *KMT2A*r, as 47% of their t(6;11) patients presented with *KRAS*-mut while only 3% of the t(9;11) had *KRAS*-mut. By contrast, in our cohort, *KRAS*-mut distribution was similar among the different translocations, with a prevalence of 19.5% for the global cohort and 24.2% for t(9;11) patients. Given the negative impact of *KRAS*-mut, this could explain the more favorable outcome of the t(9;11) cases reported in the Bill et al. study. In line with our observations, Grossman et al. [17] report a prevalence of 22.9% *KRAS*-mut in t(9;11) patients and they were also not able to find differences in OS for the distinct fusion partners. Therefore, the heterogeneous results reported in the literature with regard to a more favorable outcome of t(9;11) might be due to a potential bias in the distribution of *KRAS* mutations (rather than the fusion partner per se), although further studies will be required in order to confirm our hypothesis.

As genomic features have been described as the most powerful predictors of OS in adult AML and the interaction of genomic aberrations has been linked to outcome [3], it is reasonable to think that additional cytogenetic aberrations or concomitant gene mutations might also play a role in *KMT2A*r AML. In our study, 40% of *KMT2A*r patients presented with additional cytogenetic abnormalities resulting in higher early-death rates, although poorer long-term outcome was only associated with cases with more complex genomic alterations. While both observations need to be confirmed in independent cohort, they definitely point to additional heterogeneity within *KMT2A*r-driven leukemia biology that needs to be further understood.

In accordance, comprehensive molecular analysis of the HARMONY *KMT2A*r adult AML cohort, which to the best of our knowledge is the largest cohort studied by panel sequencing to date, revealed additional heterogeneity on the molecular level. In line with previous studies [10, 14, 17, 19], we observed a relatively low incidence of gene mutations, with a median of only one mutation per patient. This is in contrast to the average amount of additional driver mutations seen in other AML subtypes [3], most likely due to the fact that *KMT2A* is one of the strongest oncogenic drivers and is sufficient to transform healthy hematopoietic stem cells in murine models as a single molecular lesion [33].

With regard to additional oncogenic drivers, *NRAS* was the most frequently mutated gene (21% of the patients). Overall, we found a high incidence (42.1%) of gene mutations involving the *RAS* signaling pathway (*NRAS*, *KRAS, PTPN11* and *BRAF*), which is consistent with previous reports [14, 17, 34]. Furthermore, we found constitutive activation of *FLT3* by mutations in 19.1% of cases, with TKD mutations being more frequent than ITD aberrations. This is in line with a previous report in childhood *KMT2A*r leukemia that also reported more TKD than ITD mutations [35]. Of note, *NPM1* mutations were detected only in 1% of the cohort. While this constitutes a different mutational landscape in *KMT2A*r adult AML when compared to that described for the rest of adult AML [1–3], additional drivers do contribute to *KMT2A*r leukemia pathogenesis and their impact has to be further unraveled.

With regard to outcome, we were able to demonstrate that adult patients with *KMT2A*r AML have shorter OS when they present with a *KRAS* mutation. *KRAS* mutations have recently been described as an independent adverse prognostic factor in pediatric *KMT2A*r AML by the Japanese Pediatric Leukemia/Lymphoma Study Group, which was validated using a smaller cohort of adult *KMT2A*r patients [36]. However, several studies of *KMT2A*r adult AML were not able to find differences in OS for *KRAS*-mut patients, which is most likely due to the relatively small sample size and a potential bias in the distribution of *KRAS*-mut [14, 17, 19]. While *TP53*-mut have been described as an adverse prognostic marker in a univariate OS analysis in one previous study [17], we also observed an unfavorable impact of this tumor suppressor gene mutation.

The importance of gene mutations was confirmed in a subset of patients aged <60 years at diagnosis receiving intensive chemotherapy regimens, with *KRAS* and *TP53* being the most relevant genes for patient outcomes. Only 50% of the patients with either *KRAS*-mut or *TP53-*mut achieved CRc, and for those who did, median RFS was only 6 months, which resulted in a significantly shorter OS when compared to the rest of the patients of the subset. Previous studies have shown that *KRAS* mutations are likely subclonal and therefore relatively late events in *KMT2A*r AML [9, 14, 17, 34]. However, they might promote disease progression and clonal expansion of *KMT2A*r cells, as reported in a recent study using a retroviral mouse AML model [37], which could explain the low rate of treatment responses and early relapses that we observed in our patients.

On the other hand, allo-HSCT in CR might help overcome the poor prognostic impact of these additional driver mutations. The effect of allo-HSCT for *KMT2A*r AML patients achieving CR has been explored in several studies, favoring the performance of allo-HSCT whenever feasible to improve patient outcomes [19, 20, 22]. We were able to confirm these findings in our cohort, showing the benefit of allo-HSCT in CR1. In fact, our results suggest that allo-HSCT could mitigate the deleterious effect of *KRAS*-mut, *TP53-*mut and *DNMT3A*-mut, as mutated patients that underwent allo-HSCT had similar OS and RFS when compared to unmutated patients.

However, the prognosis of adult *KMT2A*r AML still remains poor, especially in patients who are not able to receive an allo-HSCT. While the development of menin inhibitors shows encouraging results in early-phase clinical trials and could change the treatment landscape of *KMT2A*r AML in the future [38, 39], *KRAS* inhibitors that have been recently approved for advanced solid tumors [40] might also open novel possibilities of targeted AML therapy and might change the prognostic impact of the markers presented in this study.

In conclusion, our study reveals a different mutational landscape of *KMT2A*r adult AML, which has important prognostic and therapeutic implications. Future studies will have to continue to better understand the disease biology underlying *KMT2A*r leukemia, especially to better guide not only conventional but also novel targeted treatment approaches and further improve patient outcome.

### Supplementary information

Supplementary Appendix

## Data Availability

After the publication of this article, data collected for this analysis and related documents will be made available to others upon reasonably justified request, which needs to be written and addressed to the attention of the corresponding author LB at the following e-mail address: lars.bullinger@charite.de. The HARMONY Alliance, via the corresponding author LB, is responsible to evaluate and eventually accept or refuse every request to disclose data and their related documents, in compliance with the ethical approval conditions, in compliance with applicable laws and regulations, and in conformance with the agreements in place with the involved subjects, the participating institutions, and all the other parties directly or indirectly involved in the participation, conduct, development, management and evaluation of this analysis.
