# Supplementary material for: Rearrangements involving 11q23.3/KMT2A in adult AML: mutational landscape and prognostic implications – a HARMONY study
Source: Leukemia. 2024 Jul 4;38(9):1929–37. doi: 10.1038/s41375-024-02333-4 (PMC11347382; doi:10.1038/s41375-024-02333-4)
Supplement: Supplementary file 1 — Supplementary Appendix [file 41375_2024_2333_MOESM1_ESM.pptx]

## Slide 1
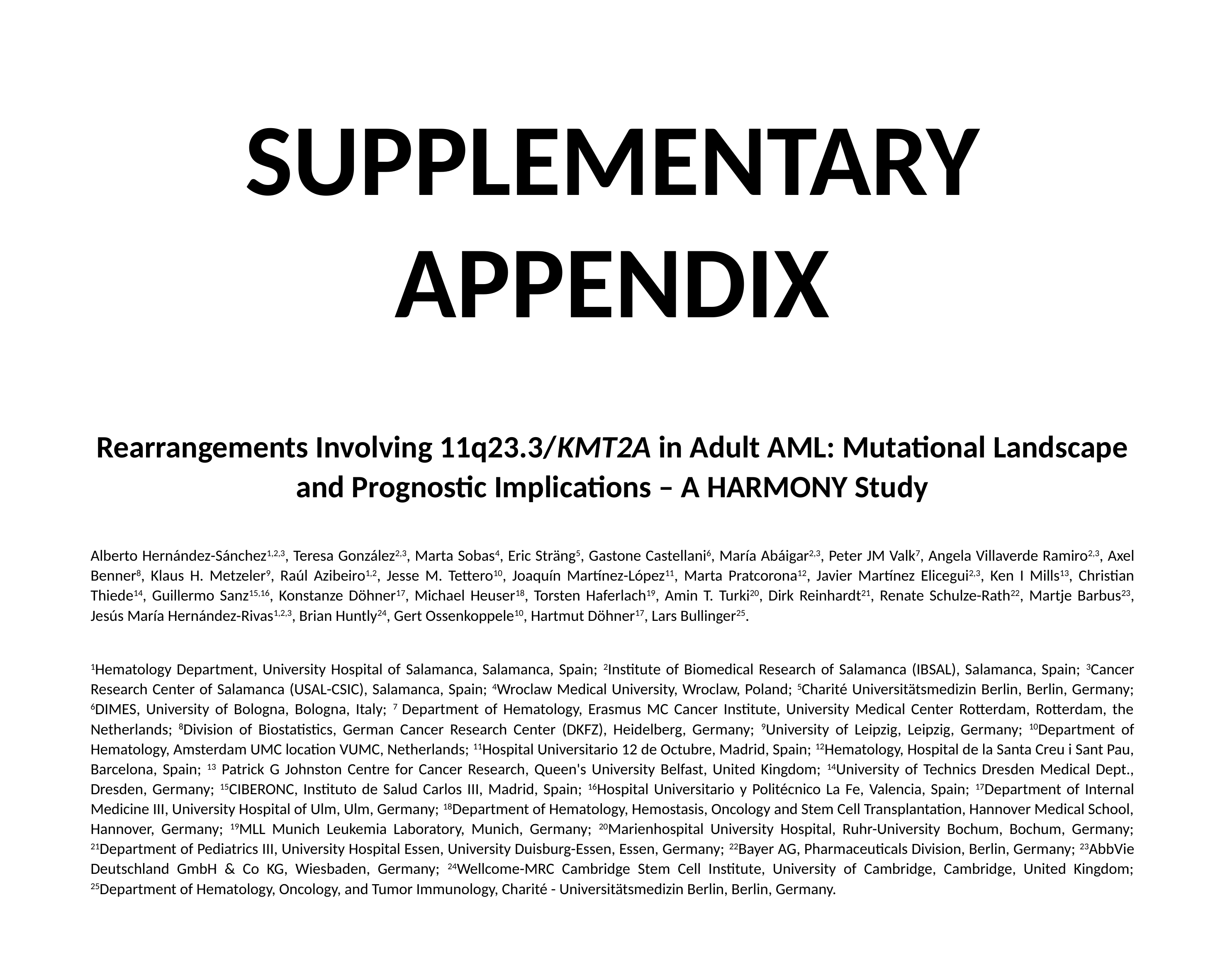

SUPPLEMENTARY
APPENDIX
Rearrangements Involving 11q23.3/KMT2A in Adult AML: Mutational Landscape and Prognostic Implications – A HARMONY Study
Alberto Hernández-Sánchez1,2,3, Teresa González2,3, Marta Sobas4, Eric Sträng5, Gastone Castellani6, María Abáigar2,3, Peter JM Valk7, Angela Villaverde Ramiro2,3, Axel Benner8, Klaus H. Metzeler9, Raúl Azibeiro1,2, Jesse M. Tettero10, Joaquín Martínez-López11, Marta Pratcorona12, Javier Martínez Elicegui2,3, Ken I Mills13, Christian Thiede14, Guillermo Sanz15,16, Konstanze Döhner17, Michael Heuser18, Torsten Haferlach19, Amin T. Turki20, Dirk Reinhardt21, Renate Schulze-Rath22, Martje Barbus23, Jesús María Hernández-Rivas1,2,3, Brian Huntly24, Gert Ossenkoppele10, Hartmut Döhner17, Lars Bullinger25.
1Hematology Department, University Hospital of Salamanca, Salamanca, Spain; 2Institute of Biomedical Research of Salamanca (IBSAL), Salamanca, Spain; 3Cancer Research Center of Salamanca (USAL-CSIC), Salamanca, Spain; 4Wroclaw Medical University, Wroclaw, Poland; 5Charité Universitätsmedizin Berlin, Berlin, Germany; 6DIMES, University of Bologna, Bologna, Italy; 7 Department of Hematology, Erasmus MC Cancer Institute, University Medical Center Rotterdam, Rotterdam, the Netherlands; 8Division of Biostatistics, German Cancer Research Center (DKFZ), Heidelberg, Germany; 9University of Leipzig, Leipzig, Germany; 10Department of Hematology, Amsterdam UMC location VUMC, Netherlands; 11Hospital Universitario 12 de Octubre, Madrid, Spain; 12Hematology, Hospital de la Santa Creu i Sant Pau, Barcelona, Spain; 13 Patrick G Johnston Centre for Cancer Research, Queen's University Belfast, United Kingdom; 14University of Technics Dresden Medical Dept., Dresden, Germany; 15CIBERONC, Instituto de Salud Carlos III, Madrid, Spain; 16Hospital Universitario y Politécnico La Fe, Valencia, Spain; 17Department of Internal Medicine III, University Hospital of Ulm, Ulm, Germany; 18Department of Hematology, Hemostasis, Oncology and Stem Cell Transplantation, Hannover Medical School, Hannover, Germany; 19MLL Munich Leukemia Laboratory, Munich, Germany; 20Marienhospital University Hospital, Ruhr-University Bochum, Bochum, Germany; 21Department of Pediatrics III, University Hospital Essen, University Duisburg-Essen, Essen, Germany; 22Bayer AG, Pharmaceuticals Division, Berlin, Germany; 23AbbVie Deutschland GmbH & Co KG, Wiesbaden, Germany; 24Wellcome-MRC Cambridge Stem Cell Institute, University of Cambridge, Cambridge, United Kingdom; 25Department of Hematology, Oncology, and Tumor Immunology, Charité - Universitätsmedizin Berlin, Berlin, Germany.

## Slide 2
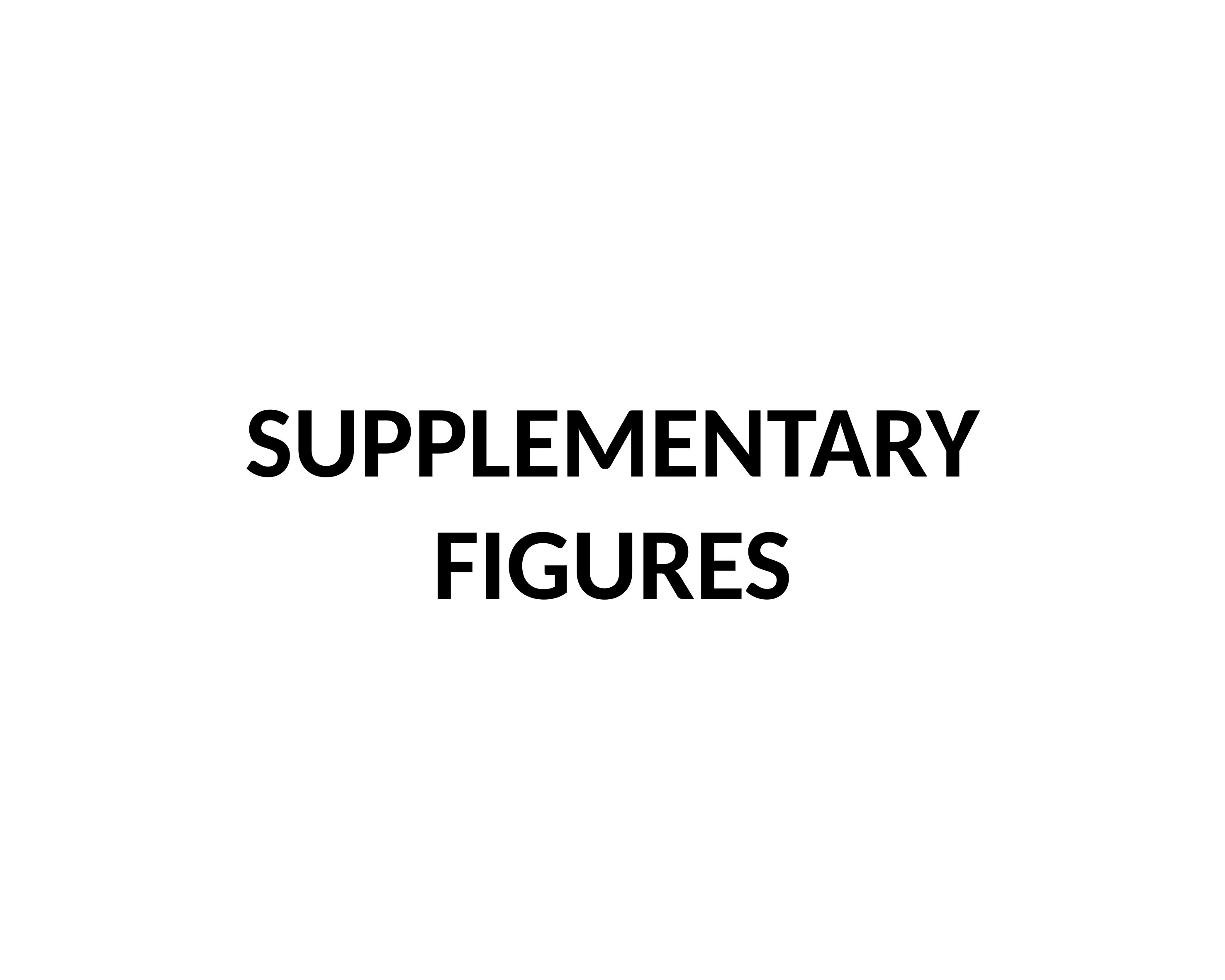

SUPPLEMENTARY
FIGURES

## Slide 3
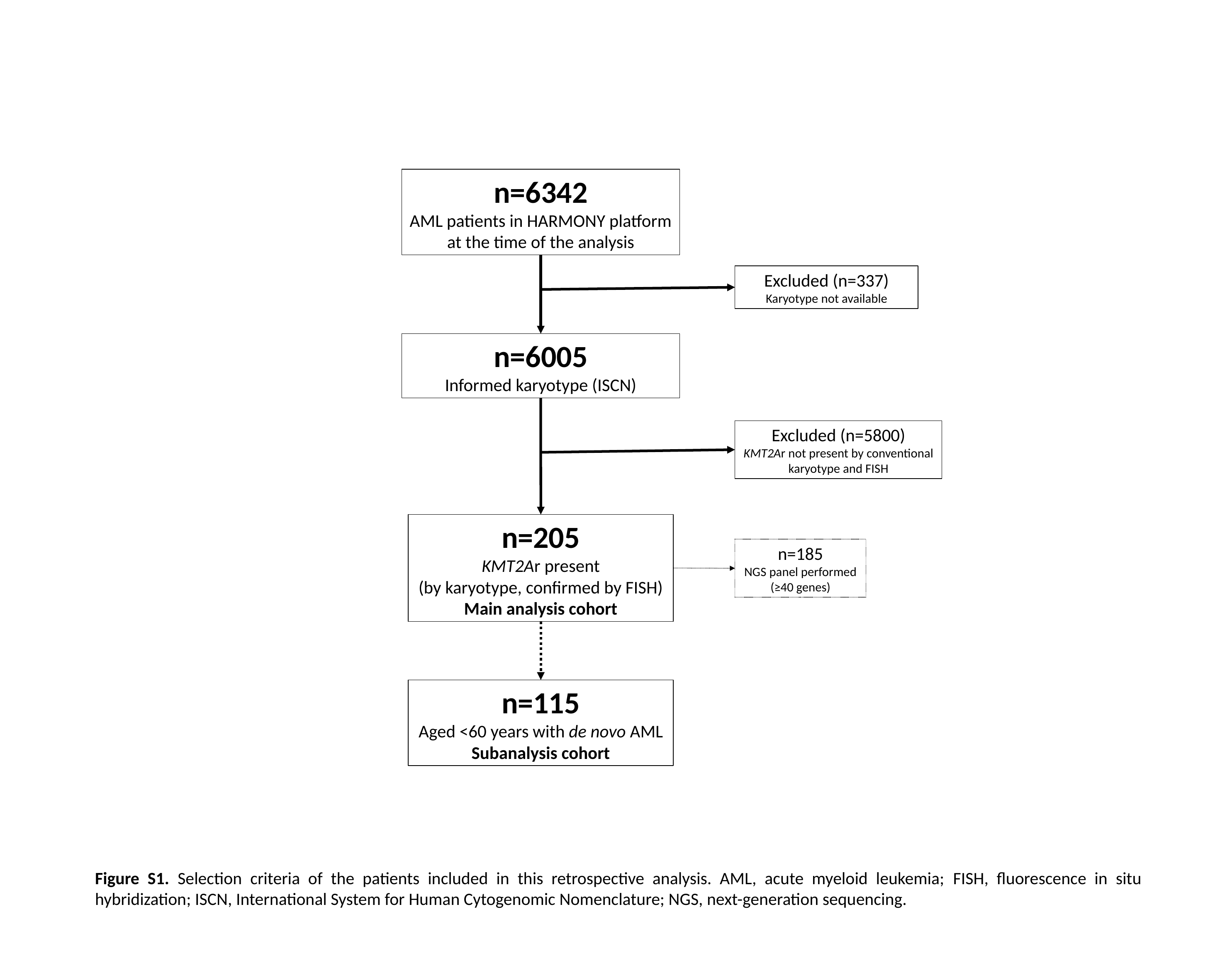

n=6342
AML patients in HARMONY platform at the time of the analysis
Excluded (n=337)
Karyotype not available
n=6005
Informed karyotype (ISCN)
Excluded (n=5800)
KMT2Ar not present by conventional karyotype and FISH
n=205
KMT2Ar present
(by karyotype, confirmed by FISH)
Main analysis cohort
n=185
NGS panel performed
(≥40 genes)
n=115
Aged <60 years with de novo AML
Subanalysis cohort
Figure S1. Selection criteria of the patients included in this retrospective analysis. AML, acute myeloid leukemia; FISH, fluorescence in situ hybridization; ISCN, International System for Human Cytogenomic Nomenclature; NGS, next-generation sequencing.

## Slide 4
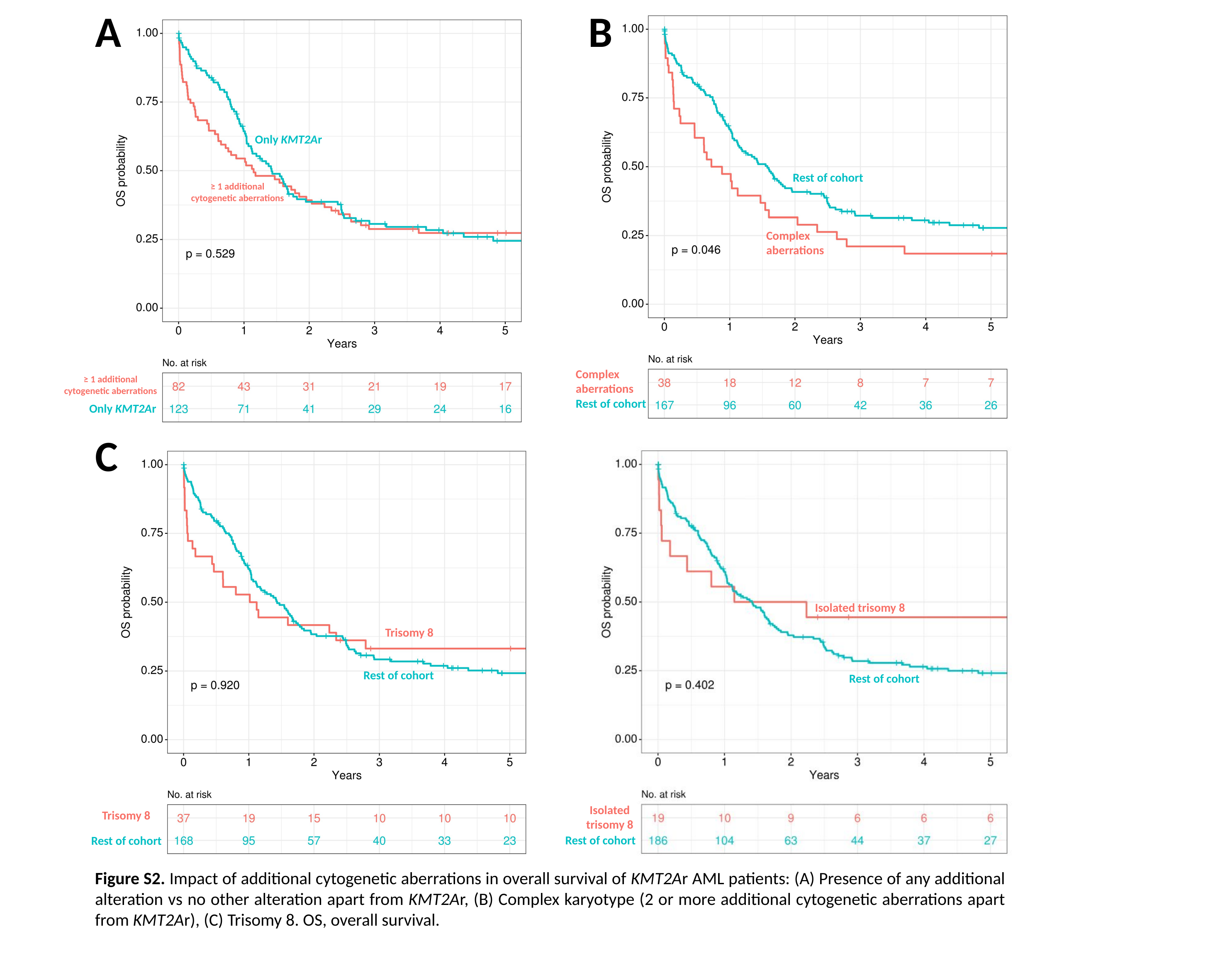

A
B
Only KMT2Ar
Rest of cohort
≥ 1 additional cytogenetic aberrations
Complex aberrations
Complex aberrations
≥ 1 additional cytogenetic aberrations
Rest of cohort
Only KMT2Ar
C
Isolated trisomy 8
Trisomy 8
Rest of cohort
Rest of cohort
Isolated trisomy 8
Trisomy 8
Rest of cohort
Rest of cohort
Figure S2. Impact of additional cytogenetic aberrations in overall survival of KMT2Ar AML patients: (A) Presence of any additional alteration vs no other alteration apart from KMT2Ar, (B) Complex karyotype (2 or more additional cytogenetic aberrations apart from KMT2Ar), (C) Trisomy 8. OS, overall survival.

## Slide 5
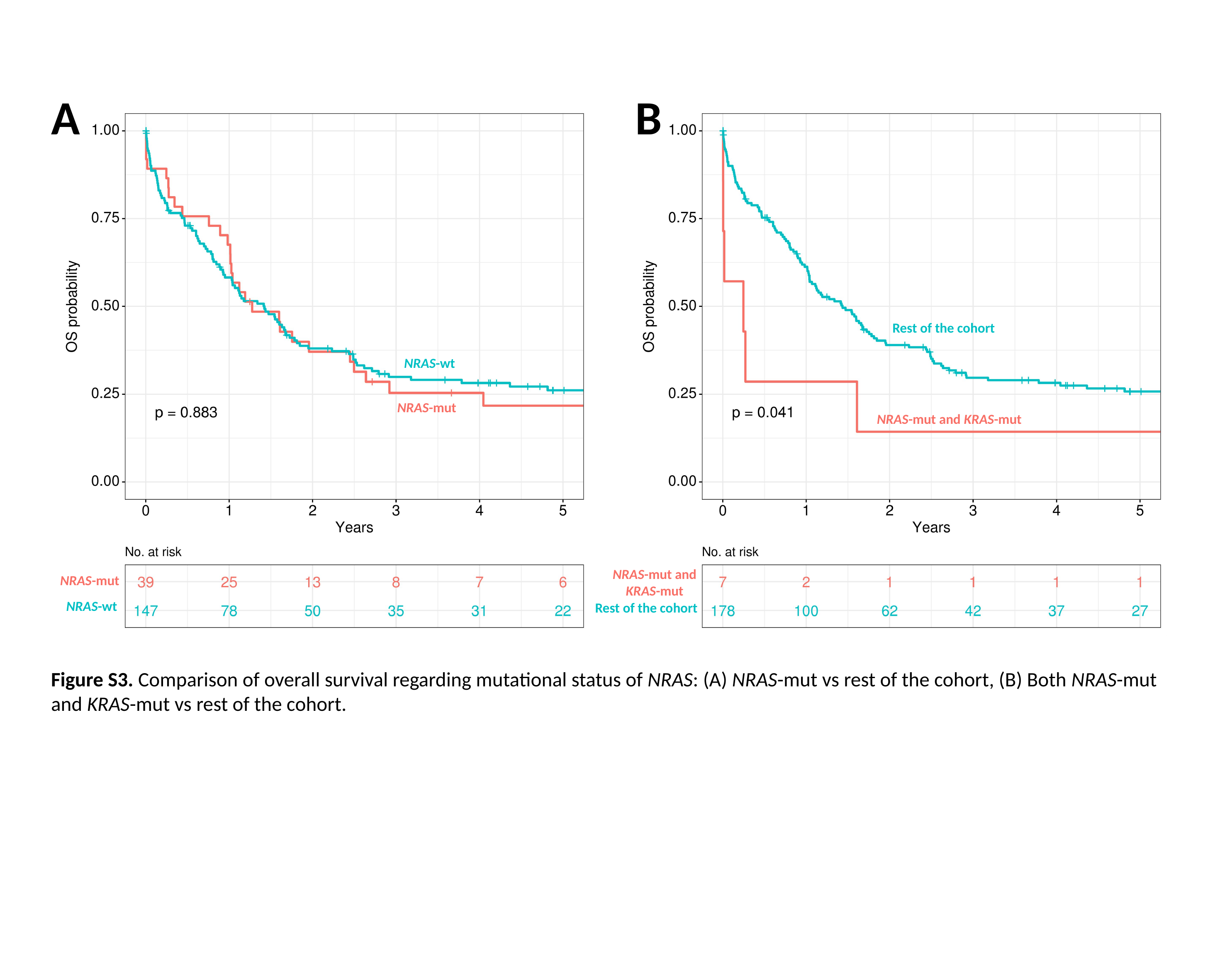

A
B
Rest of the cohort
NRAS-wt
NRAS-mut
NRAS-mut and KRAS-mut
NRAS-mut and KRAS-mut
NRAS-mut
NRAS-wt
Rest of the cohort
Figure S3. Comparison of overall survival regarding mutational status of NRAS: (A) NRAS-mut vs rest of the cohort, (B) Both NRAS-mut and KRAS-mut vs rest of the cohort.

## Slide 6
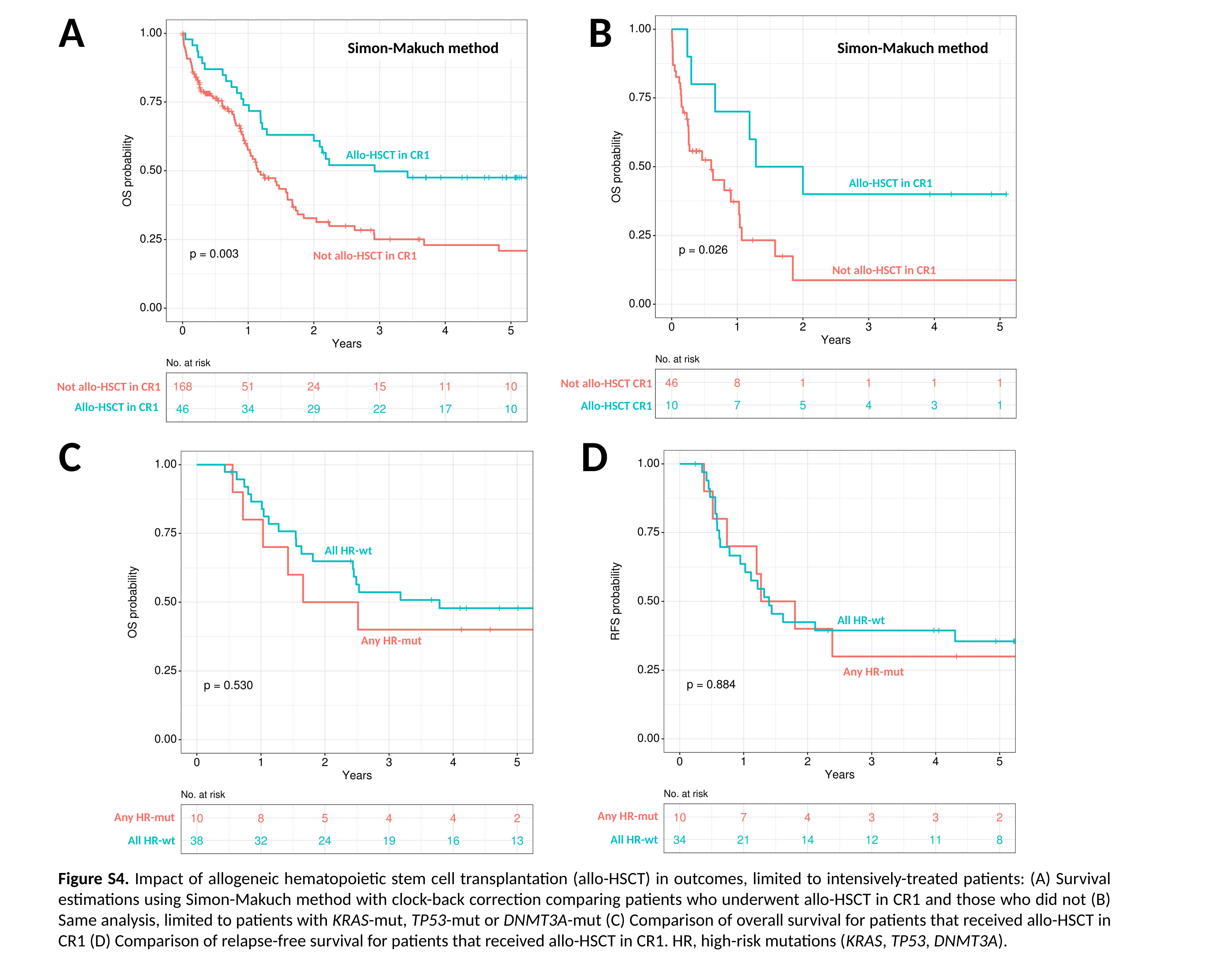

A
B
Simon-Makuch method
Simon-Makuch method
Allo-HSCT in CR1
Allo-HSCT in CR1
Not allo-HSCT in CR1
Not allo-HSCT in CR1
Not allo-HSCT CR1
Not allo-HSCT in CR1
Allo-HSCT CR1
Allo-HSCT in CR1
C
D
All HR-wt
All HR-wt
Any HR-mut
Any HR-mut
Any HR-mut
Any HR-mut
All HR-wt
All HR-wt
Figure S4. Impact of allogeneic hematopoietic stem cell transplantation (allo-HSCT) in outcomes, limited to intensively-treated patients: (A) Survival estimations using Simon-Makuch method with clock-back correction comparing patients who underwent allo-HSCT in CR1 and those who did not (B) Same analysis, limited to patients with KRAS-mut, TP53-mut or DNMT3A-mut (C) Comparison of overall survival for patients that received allo-HSCT in CR1 (D) Comparison of relapse-free survival for patients that received allo-HSCT in CR1. HR, high-risk mutations (KRAS, TP53, DNMT3A).

## Slide 7
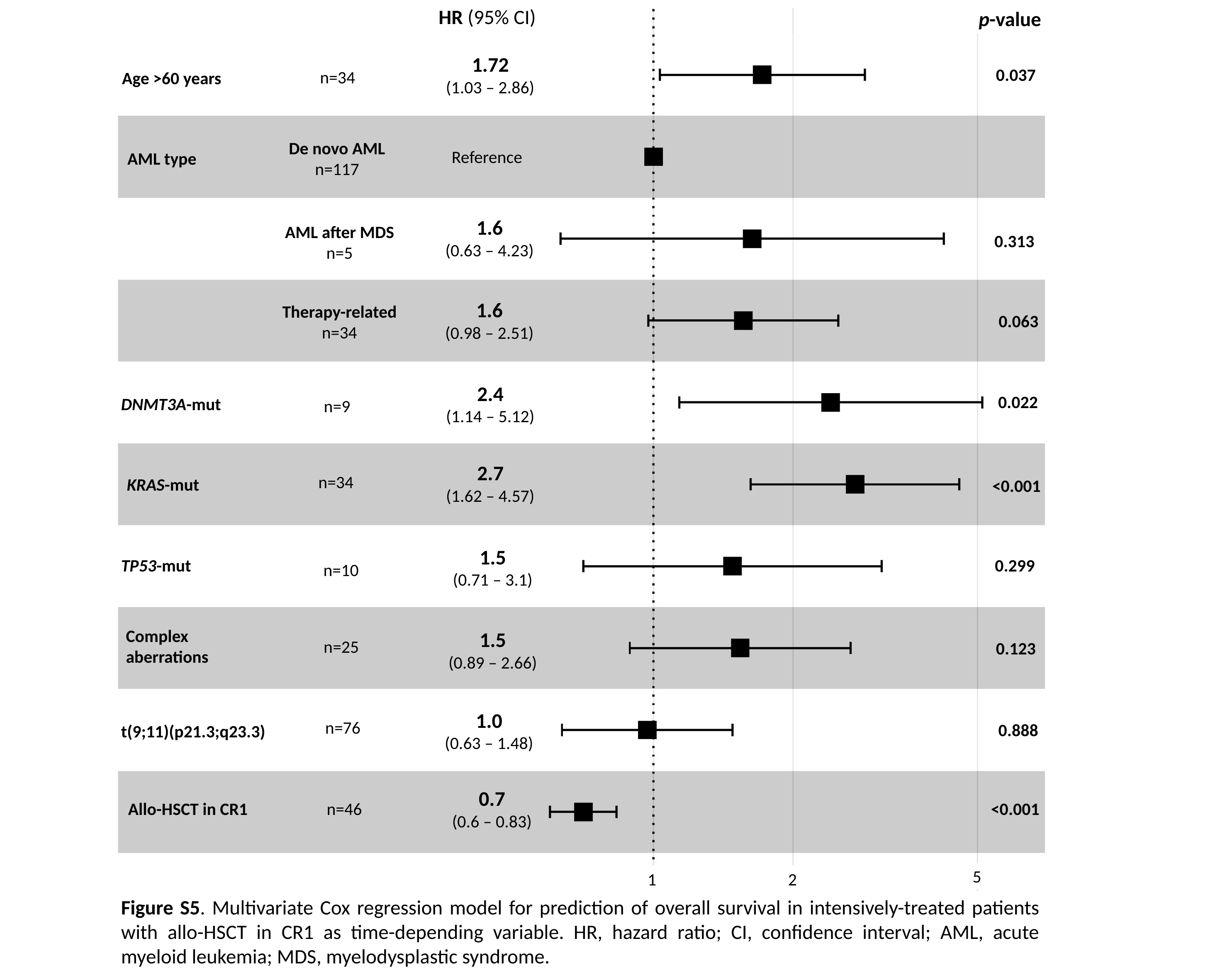

HR (95% CI)
p-value
1.72
(1.03 – 2.86)
0.037
n=34
Age >60 years
De novo AML
n=117
Reference
AML type
1.6
(0.63 – 4.23)
AML after MDS
n=5
0.313
1.6
(0.98 – 2.51)
Therapy-related
n=34
0.063
2.4
(1.14 – 5.12)
0.022
DNMT3A-mut
n=9
2.7
(1.62 – 4.57)
n=34
KRAS-mut
<0.001
1.5
(0.71 – 3.1)
0.299
TP53-mut
n=10
Complex aberrations
1.5
(0.89 – 2.66)
n=25
0.123
1.0
(0.63 – 1.48)
n=76
0.888
t(9;11)(p21.3;q23.3)
0.7
(0.6 – 0.83)
Allo-HSCT in CR1
<0.001
n=46
5
1
2
Figure S5. Multivariate Cox regression model for prediction of overall survival in intensively-treated patients with allo-HSCT in CR1 as time-depending variable. HR, hazard ratio; CI, confidence interval; AML, acute myeloid leukemia; MDS, myelodysplastic syndrome.

## Slide 8
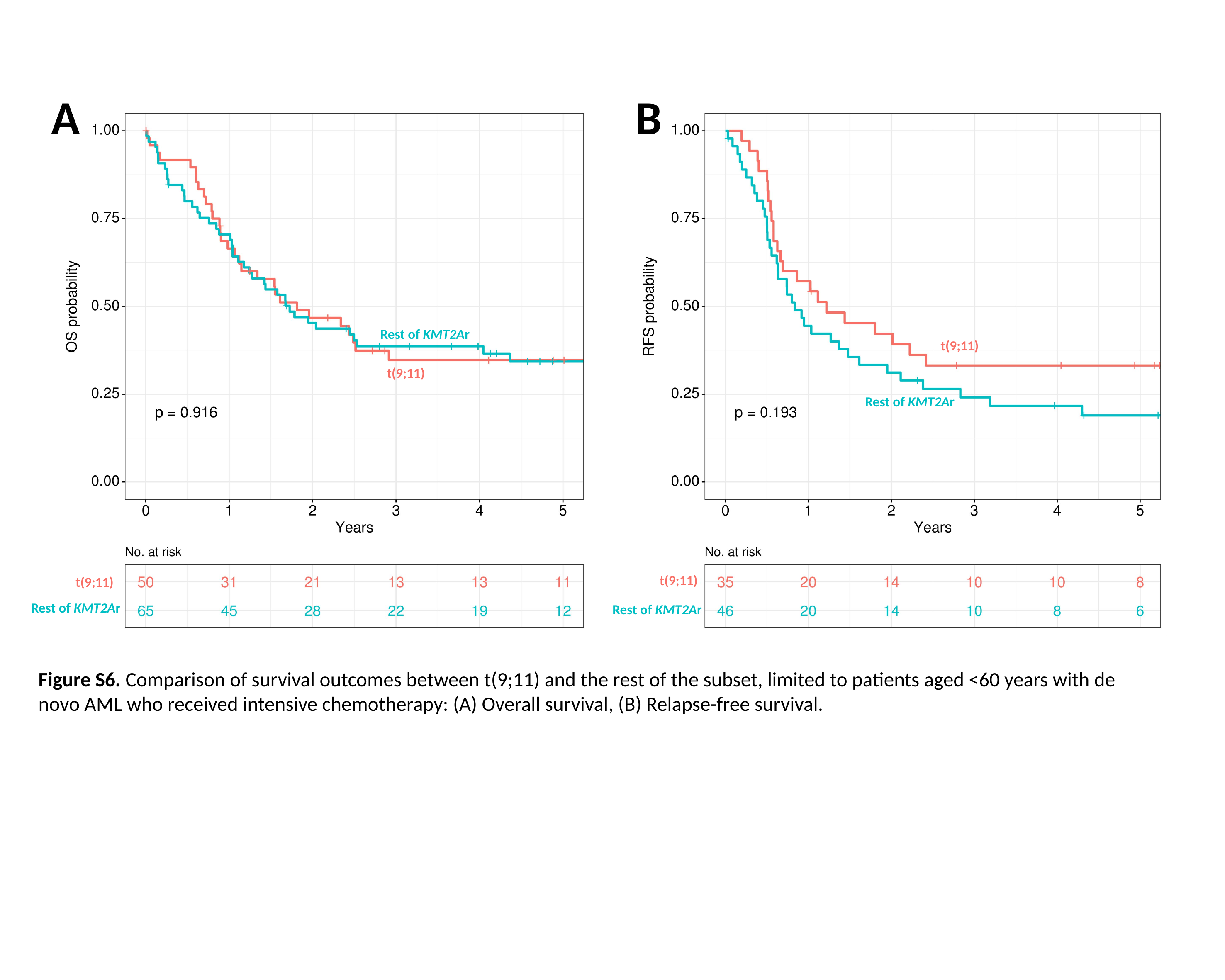

A
B
Rest of KMT2Ar
t(9;11)
t(9;11)
Rest of KMT2Ar
t(9;11)
t(9;11)
Rest of KMT2Ar
Rest of KMT2Ar
Figure S6. Comparison of survival outcomes between t(9;11) and the rest of the subset, limited to patients aged <60 years with de novo AML who received intensive chemotherapy: (A) Overall survival, (B) Relapse-free survival.

## Slide 9
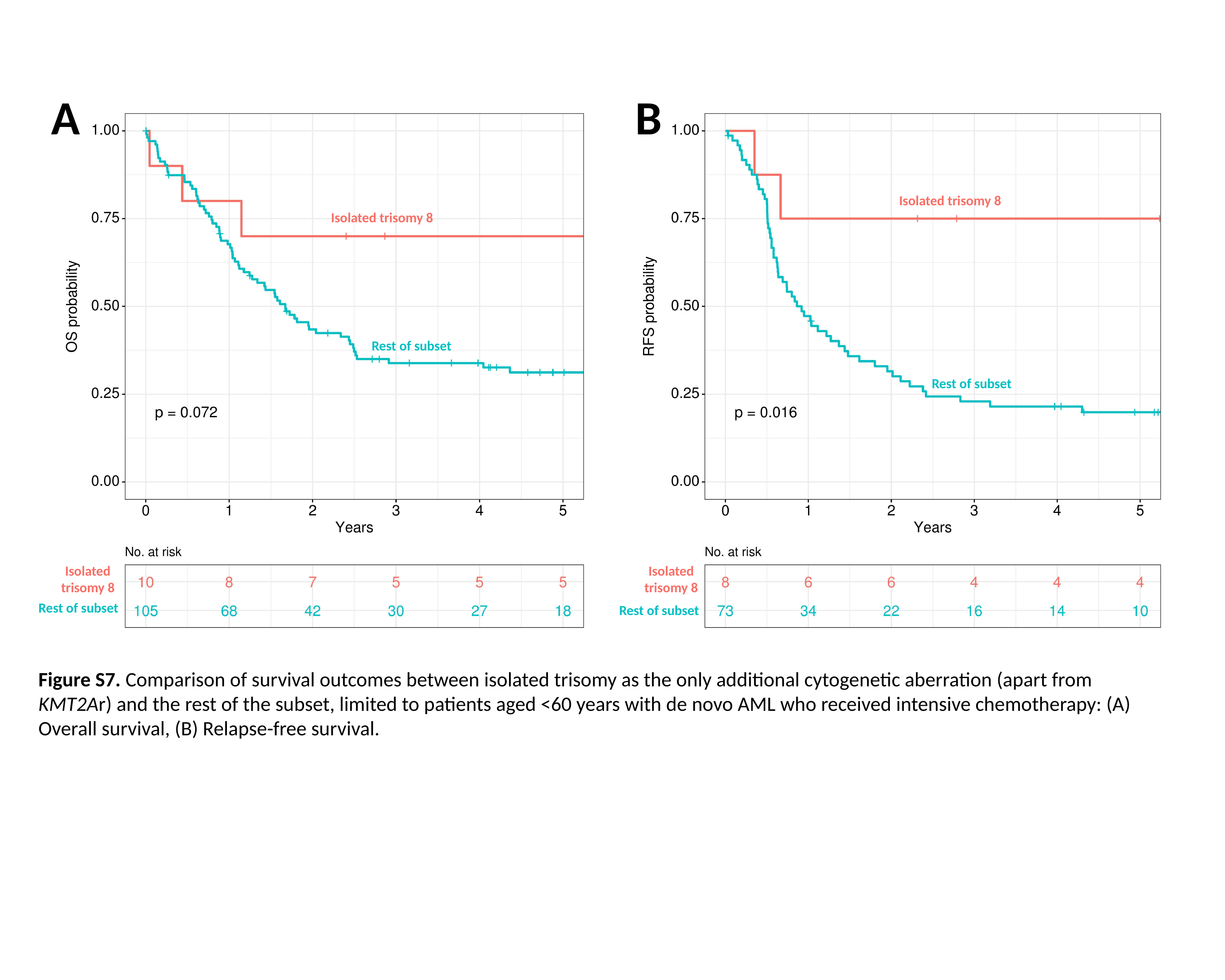

A
B
Isolated trisomy 8
Isolated trisomy 8
Rest of subset
Rest of subset
Isolated trisomy 8
Isolated trisomy 8
Rest of subset
Rest of subset
Figure S7. Comparison of survival outcomes between isolated trisomy as the only additional cytogenetic aberration (apart from KMT2Ar) and the rest of the subset, limited to patients aged <60 years with de novo AML who received intensive chemotherapy: (A) Overall survival, (B) Relapse-free survival.

## Slide 10
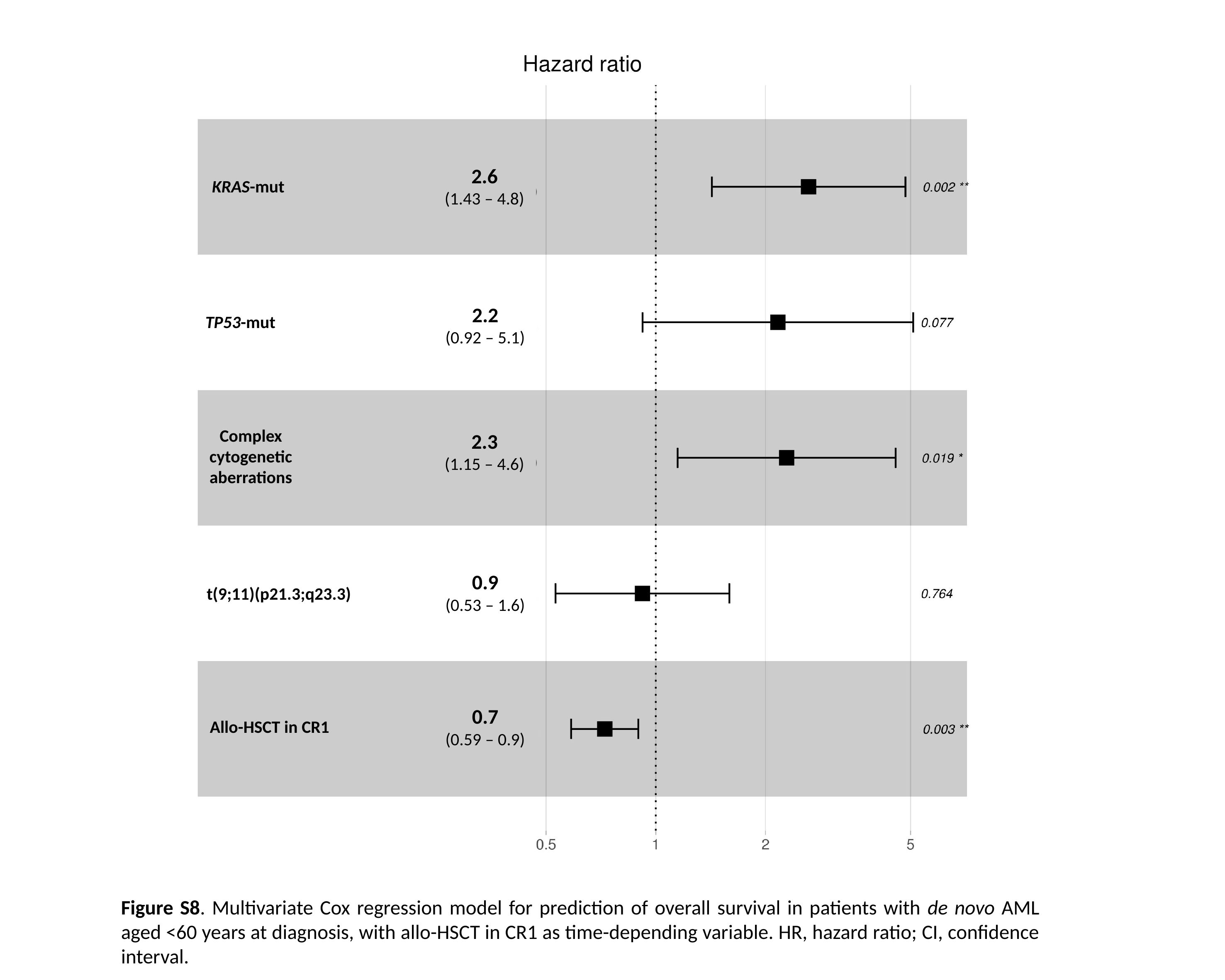

2.6
(1.43 – 4.8)
KRAS-mut
2.2
(0.92 – 5.1)
TP53-mut
Complex cytogenetic aberrations
2.3
(1.15 – 4.6)
0.9
(0.53 – 1.6)
t(9;11)(p21.3;q23.3)
0.7
(0.59 – 0.9)
Allo-HSCT in CR1
Figure S8. Multivariate Cox regression model for prediction of overall survival in patients with de novo AML aged <60 years at diagnosis, with allo-HSCT in CR1 as time-depending variable. HR, hazard ratio; CI, confidence interval.

## Slide 11
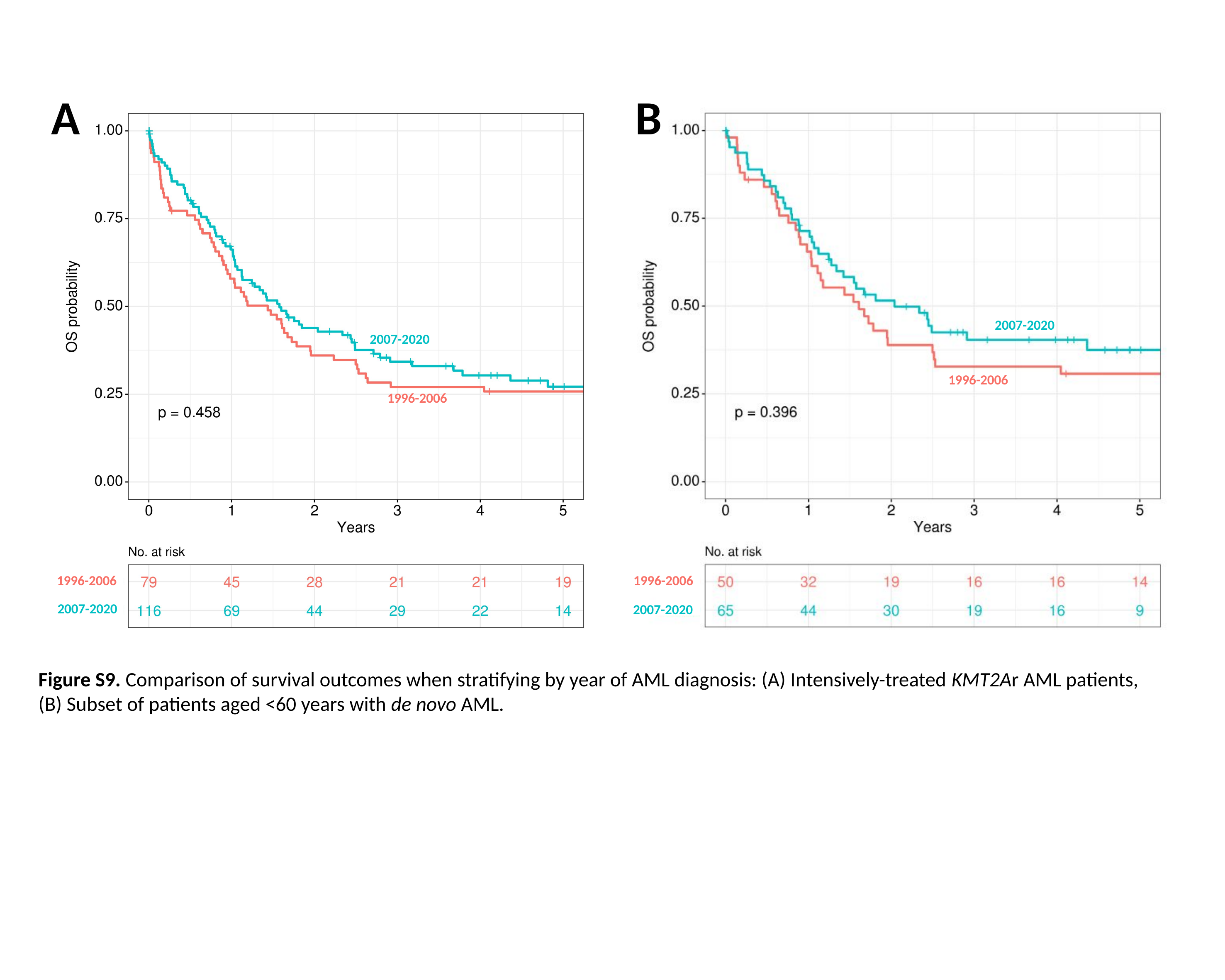

A
B
2007-2020
2007-2020
1996-2006
1996-2006
1996-2006
1996-2006
2007-2020
2007-2020
Figure S9. Comparison of survival outcomes when stratifying by year of AML diagnosis: (A) Intensively-treated KMT2Ar AML patients, (B) Subset of patients aged <60 years with de novo AML.

## Slide 12
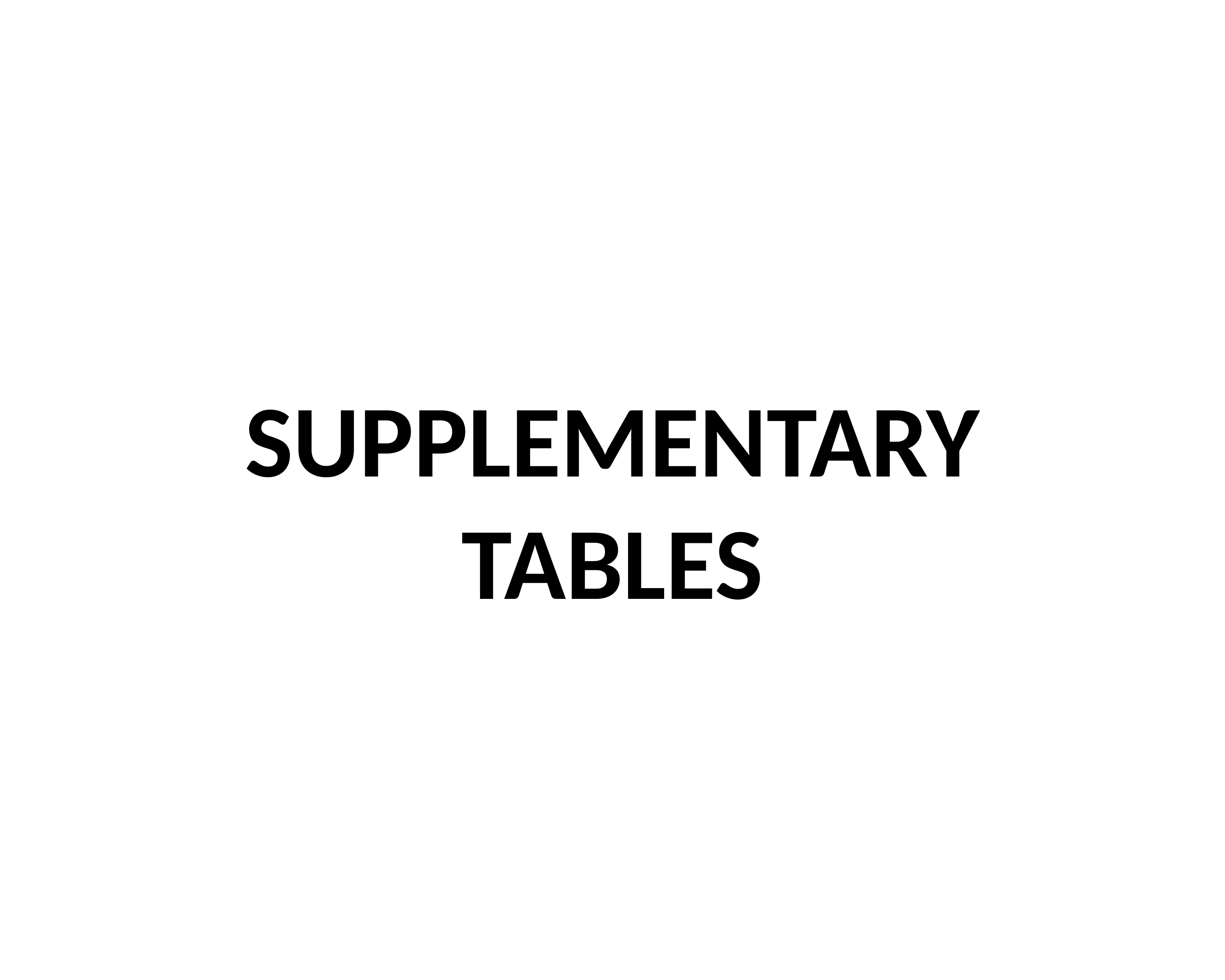

SUPPLEMENTARY
TABLES

## Slide 13
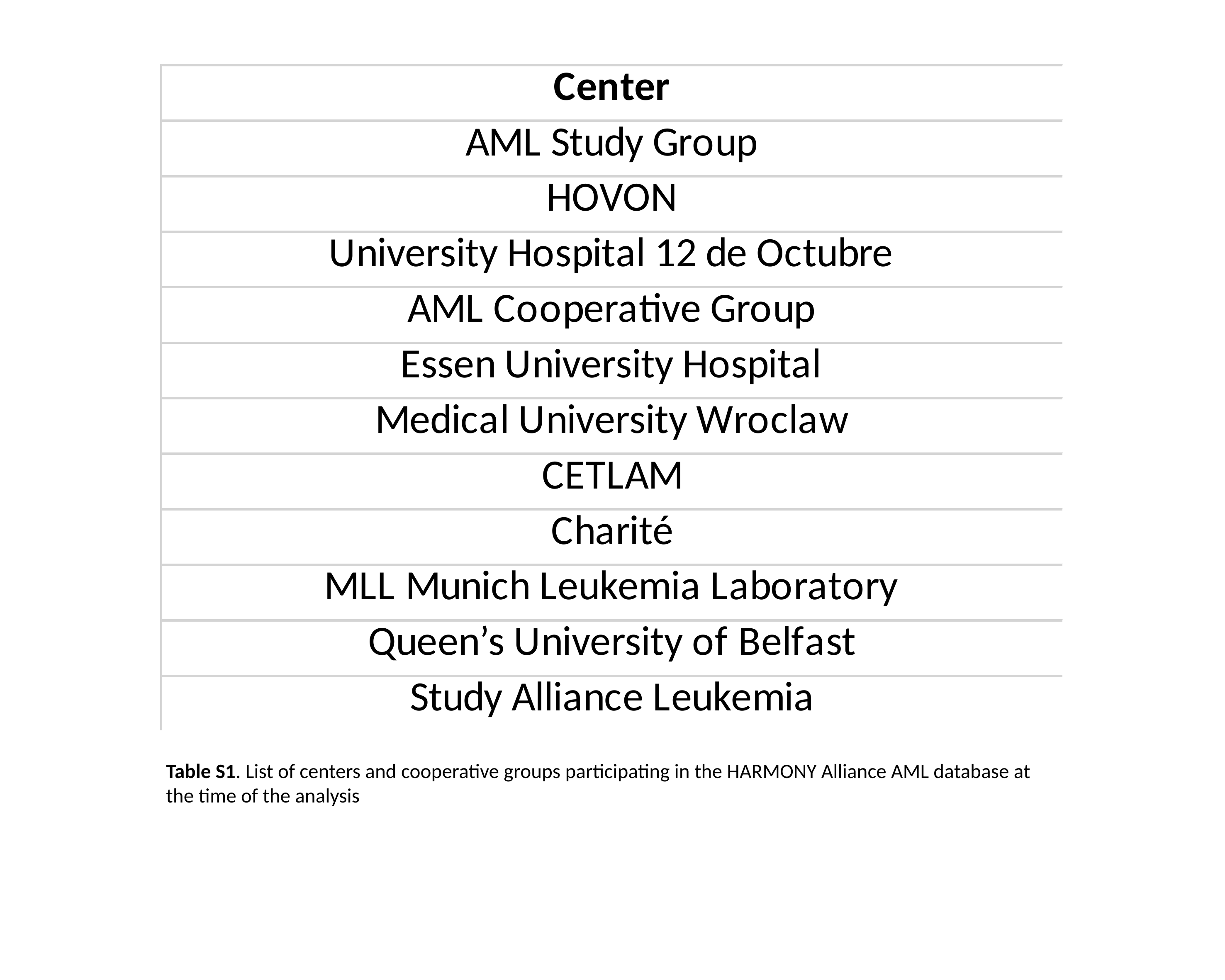

Table S1. List of centers and cooperative groups participating in the HARMONY Alliance AML database at the time of the analysis

## Slide 14
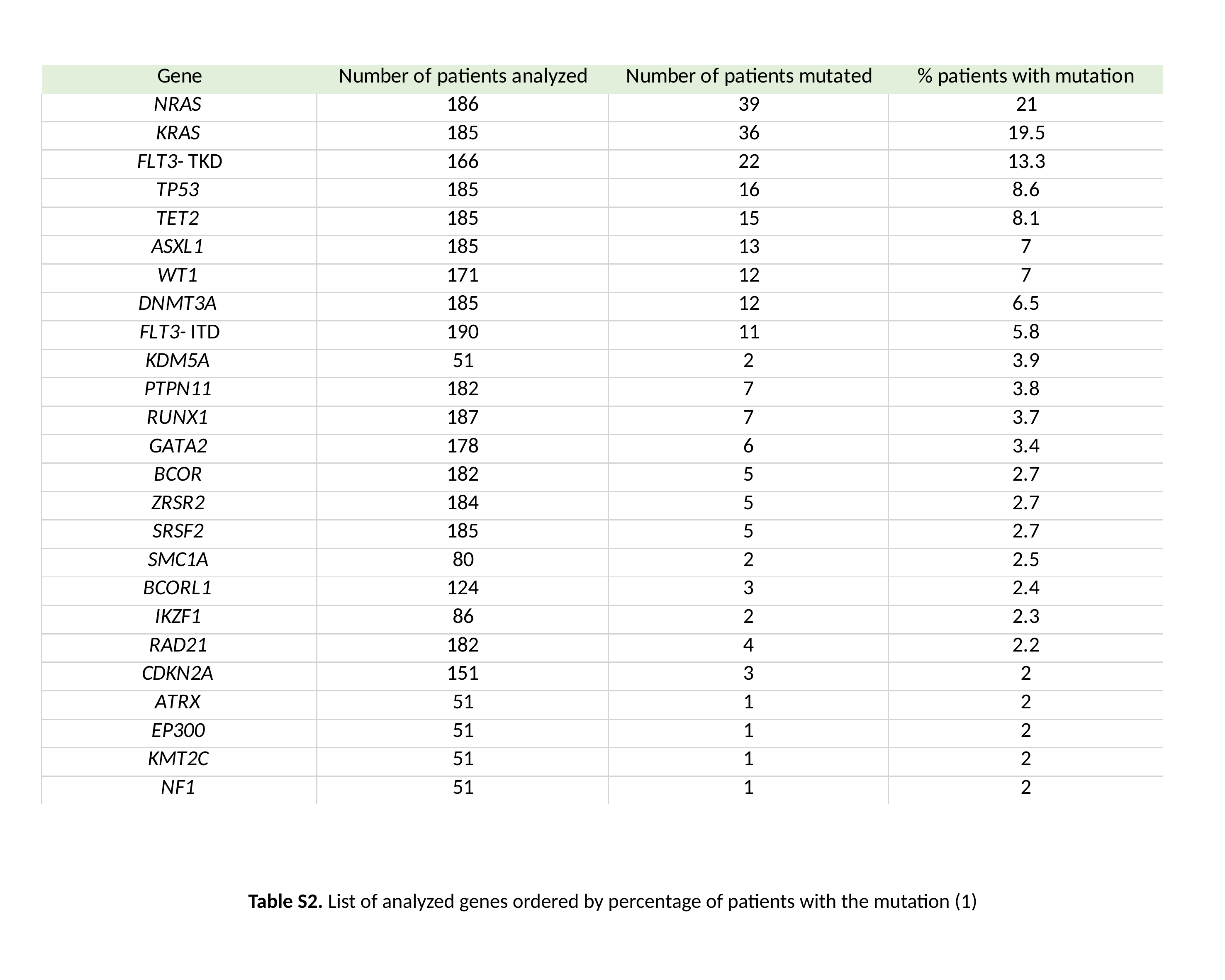

Table S2. List of analyzed genes ordered by percentage of patients with the mutation (1)

## Slide 15
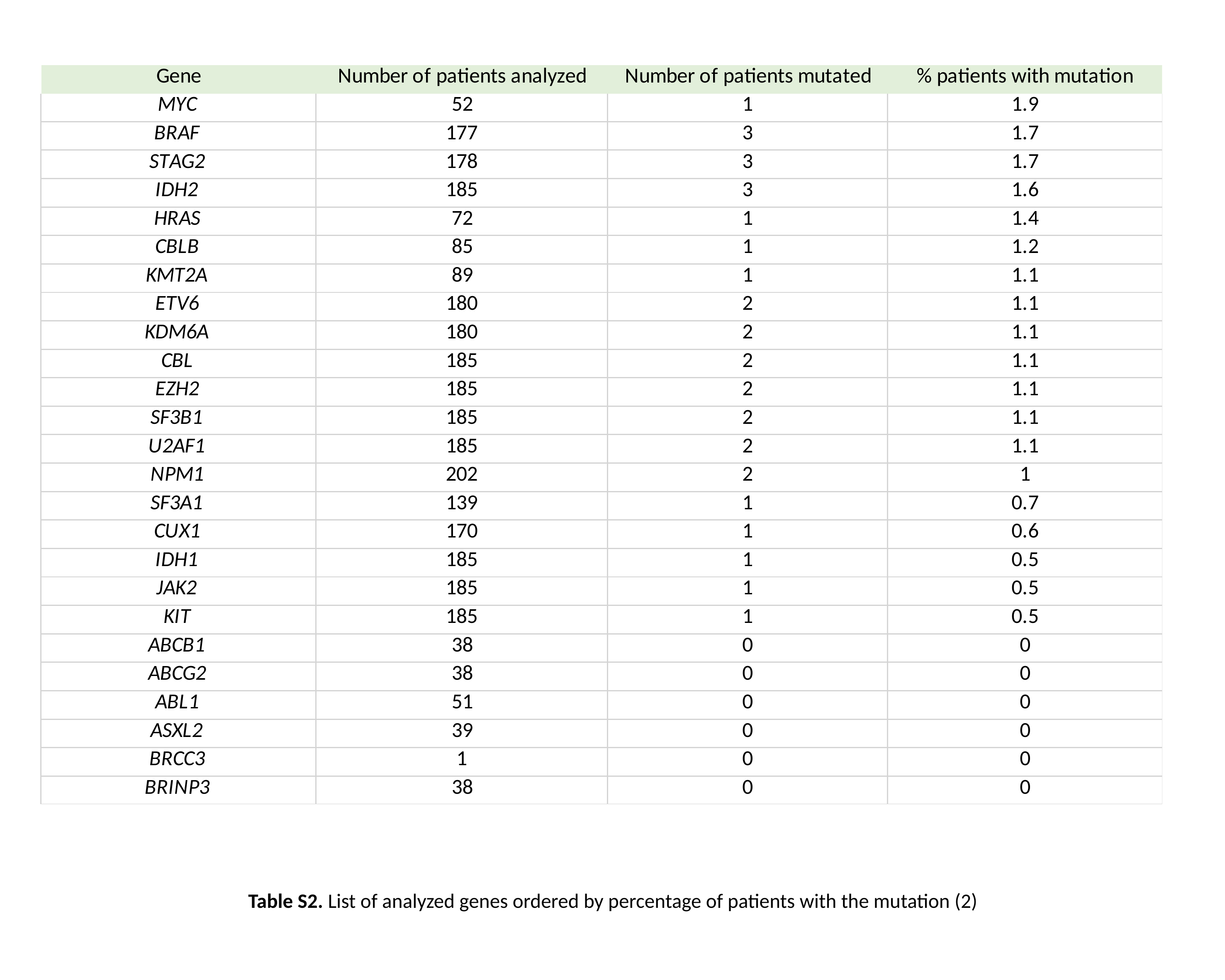

Table S2. List of analyzed genes ordered by percentage of patients with the mutation (2)

## Slide 16
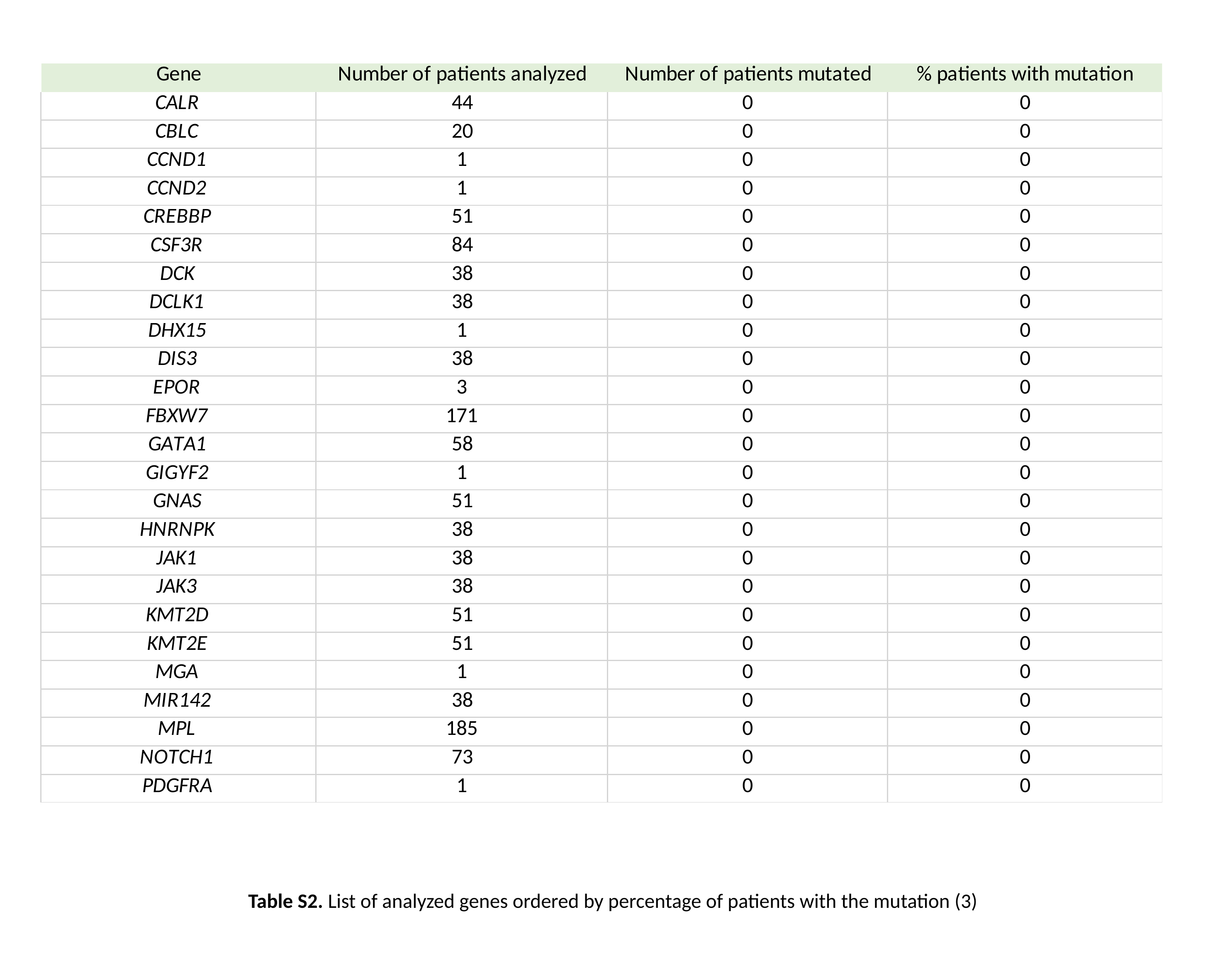

Table S2. List of analyzed genes ordered by percentage of patients with the mutation (3)

## Slide 17
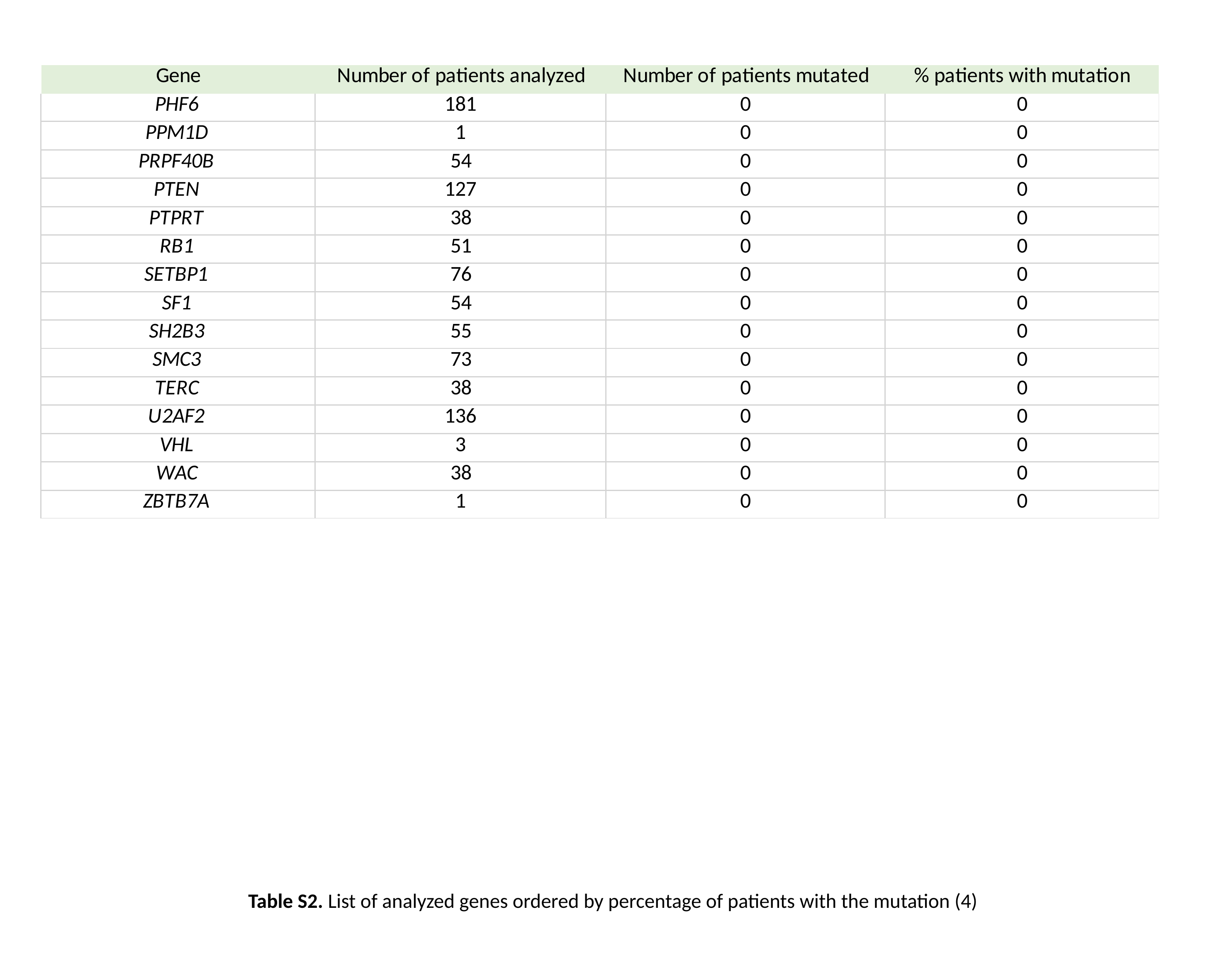

Table S2. List of analyzed genes ordered by percentage of patients with the mutation (4)

## Slide 18
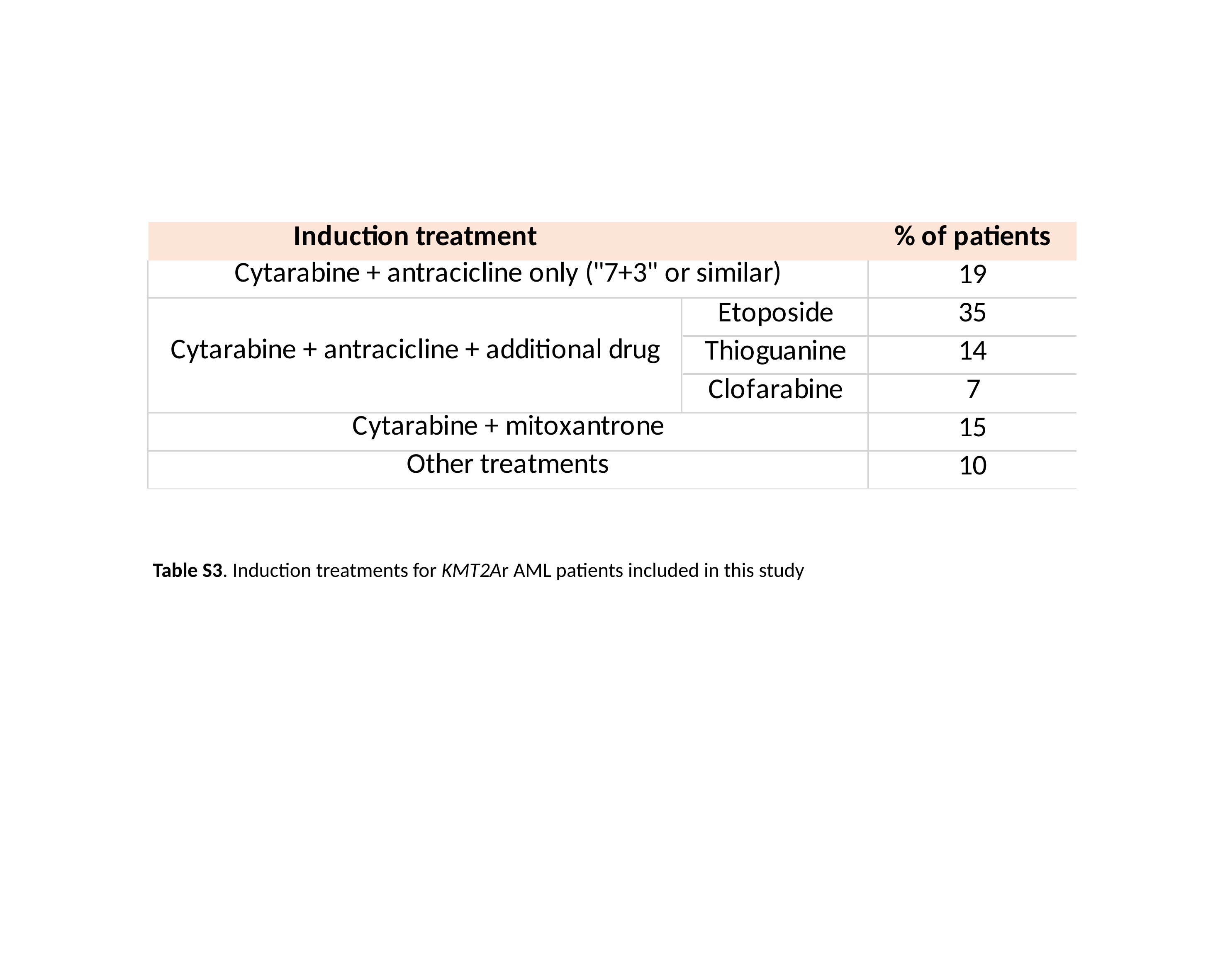

Table S3. Induction treatments for KMT2Ar AML patients included in this study

## Slide 19
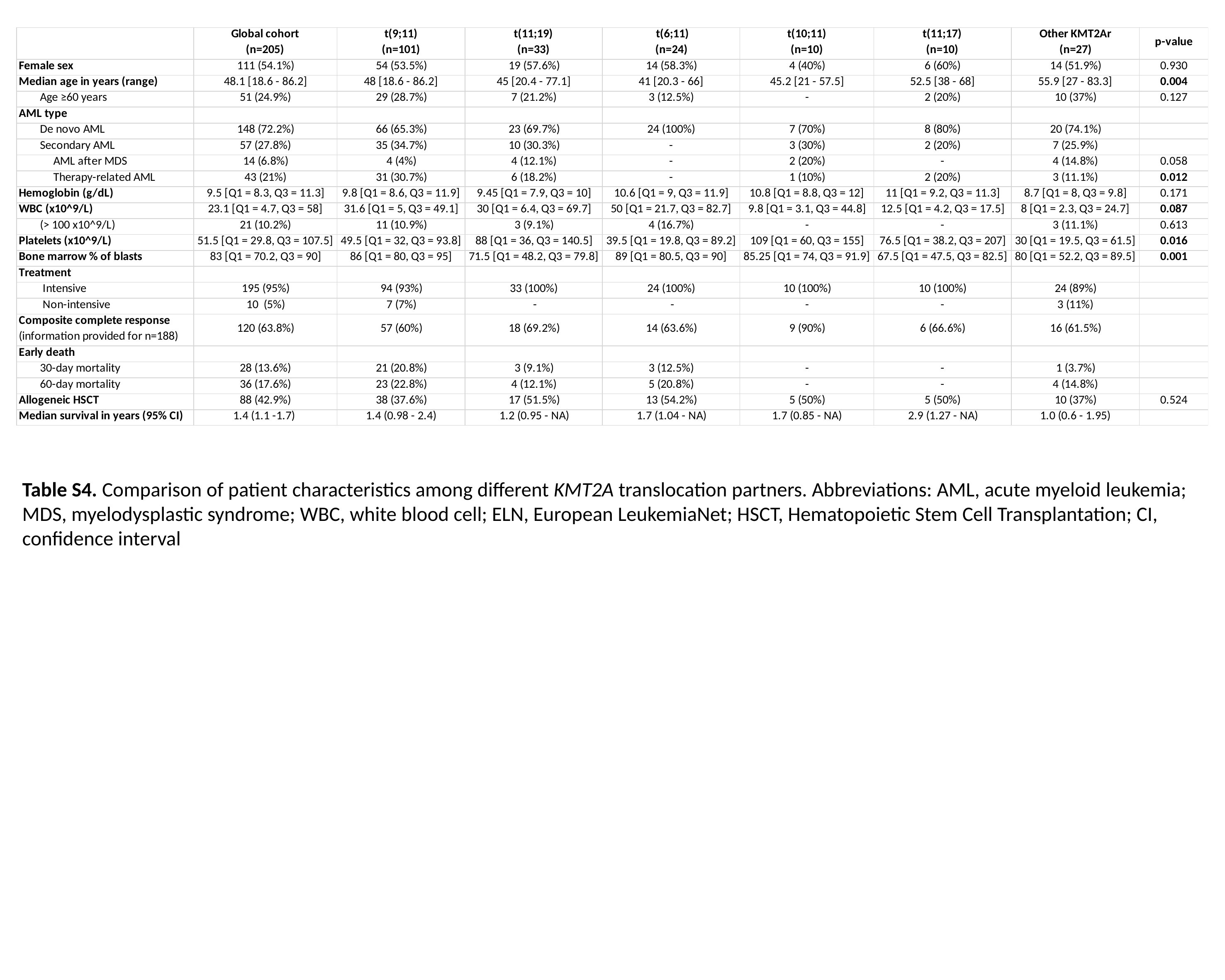

Table S4. Comparison of patient characteristics among different KMT2A translocation partners. Abbreviations: AML, acute myeloid leukemia; MDS, myelodysplastic syndrome; WBC, white blood cell; ELN, European LeukemiaNet; HSCT, Hematopoietic Stem Cell Transplantation; CI, confidence interval
